# Design of a Triple Emissive Cu_4_I_4_ Coordination Polymer through Ligand Engineering

**DOI:** 10.1021/acs.inorgchem.5c04789

**Published:** 2025-12-12

**Authors:** Léo Boivin, Daniel Fortin, Pierre D. Harvey

**Affiliations:** 7321 Département de chimie de l’Université de Sherbrooke, Sherbrooke, Quebec J1K 2R1, Canada

## Abstract

Through rational ligand design, the coordination polymer 1D-[Cu_4_(μ_3_-I)_4_(μ_2_-**L1**)_2_]_*n*_ (**CP1**; **L1** = 1,1-(1′-naphthylthio)­methane) has been predictably prepared from CuI and **L1** in acetonitrile where both the node, closed cubane Cu_4_(μ_3_-I)_4_ cluster, and the assembling ligand containing naphthyl aromatic pendants are known to be emissive. At liquid nitrogen temperature, **CP1** exhibits simultaneously three long-lived emissions centered at 573, 732, and 785 nm, decaying in the ms time scale (∼7.1 ms), which are readily assigned to triplet metal/ligand-to-ligand charge transfer (^3^ M/XLCT), ligand-centered (^3^LC; i.e., ^3^ππ*), and excimer (^3^excimer), respectively. These assignments, which strikingly diverge from the classical low-energy cluster-centered (^3^CC) emission emerging from the Cu_4_(μ_3_-I)_4_ cluster, were based on in-depth photophysical studies and advanced quantum simulations by density functional theory (DFT) and time-dependent DFT (TD-DFT), which also calculated the emission maxima with high precision and degree of agreement.

## Graphical abstract

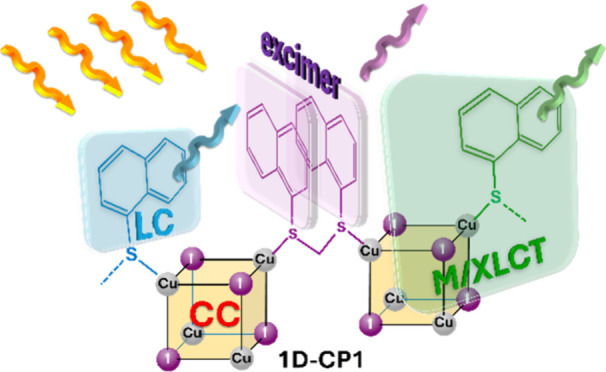

## 1 Introduction

The multiresponsive Cu_4_I_4_L_4_ closed-cubane cluster is a prime construction unit for the design of functional materials because of its large versatility of ligand types (L = N-, P-, As-, S-, Se-, Te-donors) used for the fabrication of 0D-discrete complexes and porous/nonporous 1D-, 2D-, 3D- coordination polymers (CPs), including metal–organic frameworks (MOFs)^1^ where the Cu_4_I_4_-cubane acts as the secondary building unit (SBU). Because of its resulting CP structure, its intrinsic intense luminescence and rich photophysical properties, applications such as sensors,^2–7^ X-ray phosphors and scintillators,^8,9^ adsorption, separation and capture of substrates and pollutants,^10,11^ two- or three-photon absorbers and nonlinear optical materials,^12,13^ luminescent thermometers,^14^ catalysts,^15–17^ photocatalysts,^18^ white light electroluminescent materials,^19^ hole transport layers in solar cells,^20^ and anticorrosion,^21^ and antibacterial materials,^21^ have been reported. They are also often found to be stimuli-responsive toward pressure,^22–24^ temperature,^14,25–27^ vapor,^28^ solvent,^28^ and mechanical stresses.^23,27,29^ This latter property permits to design functional coatings capable of visually detecting the presence of past mechanical shocks onto a surface.^29,30^ The possibility of forming thermochromic liquid crystals containing the Cu_4_I_4_P_4_-core was also demonstrated.^31^

Concurrently, several properties and applications of (CuX)_*n*_-containing materials (X = halide) rely on two emissions arising from energetically similar and interacting excited states, such as T_1_ and S_1_ for TADF materials used in OLEDs,^32–36^ or on the coexistence of different triplet excited states (^3^ M/XLCT and ^3^CC) for designs of white light-emitting phosphors.^37–39^ Moreover, developments have also been made where the noninnocent (Cu_2_X_2_) nodes (X = Br, I) were combined with redox-active and chromophoric ligands such as fluorenylidene malononitrile, and dithiofluorenones, opening the door to different properties such as photoconductivity and enhanced photosensitization.^40,41^

The emissive subcategory Cu_4_I_4_L_4_-containing CPs, where L is S-donor ligands such as thioethers and thiones, makes no exception and is also rich in photophysical properties^42^ and applications,^43^ which have been thoroughly reviewed. One of the most exploited types of ligands is the cyclic and acyclic dithioether RS­(CH_2_)_*m*_SR motif (R = alkyl or aryl (mainly substituted phenyl groups); *m* = 1–8)^44^ for the preparation of luminescent 1D-, 2D-, and 3D-CPs,^45–47^ as well as 2D-MOF materials,^48^ but lacks unusual photophysical properties or applications. To the best of our knowledge, only one thermally responsive and strongly luminescent CP, 1D-[Cu_4_I_4_(μ-(MeOC_6_H_4_S­(CH_2_)_4_SC_6_H_4_SC_6_H_4_OMe)­(N≡CMe)_2_]_*n*_, which irreversibly undergoes a chemical transformation (upon the evaporation of the acetonitrile ligands) to form a poorly emissive 3D-CP, has been reported so far.^49^ Such a temperature-sensitive material can potentially serve as an indicator going from emissive (0D-Cu_4_I_4_ SBU) to a nonemissive (1D-Cu_8_I_8_ SBU) at room temperature if the material has been exposed to a temperature exceeding a given value (in this case, >78 °C). In brief, the structural simplicity of the current RS­(CH_2_)_*m*_SR motifs strongly precludes opportunities to design materials with new, original optical properties and functionality.

To remedy this situation, stimuli-responsive groups can be used to replace any spectator segments, such as the (CH_2_)_*m*_-chain^41^ or R pendants. Thus, in this study, copper­(I) iodide and a naphthyl-based bridging ligand are combined. The naphthyl side-group was selected due to its ability to emit in the visible region,^50–52^ and its ability to form excimers on occasions,^50,52–55^ permitting to modulate the luminescence properties of the CP. In addition, a one-carbon −CH_2_– chain was chosen to design the ArSCH_2_SAr motif as it is prone to form Cu_4_I_4_ cubane-containing CPs with 100% probability based on 4 precedent examples (Ar = 4-C_6_H_4_X; X = H, Me, OMe, Br).^44,56,57^ Moreover, the Cu_4_I_4_S_4_ closed cubane motif systematically exhibits an intense emission from ^3^CC states at room temperature, regardless of whether it is embedded in the backbone of a CP or if it is a distinct coordination complex. Since these materials exhibit highly luminescent ^3^CC states, it is of great interest to tune the overall emission of the CP via the addition of emissive ^1,3^LC states. Unexpectedly, **CP1** is nonemissive at 293 K and weakly emissive at 77 K, where an excitation wavelength-dependent triple emission was observed. So, this new material is temperature-dependent in an “on–off” manner. However, a thorough photo-physical and theoretical study, combined with a rigorous investigation of the ligand triplet emission properties, allowed for securely assessing the photophysical traits of **CP1** ^3^MXLCT, ^3^LC, and ^3^excimer emissions, and ultimately explain the “on–off” behavior.

## 2 Experimental Section

### 2.1 Materials

All starting reagents and solvents were purchased from Sigma-Aldrich, Fisher Scientific, Oakwood Chemicals, or AA Blocks and used without further purification.

### 2.2 Synthetic Methods

#### 2.2.1 Synthesis of 1-Naphthalene Mercaptan

The intermediate 1-naphthalene mercaptan was synthesized from the commercially acquired 1-iodonaphthalene (AA Blocks) according to literature protocols^58^ and purified using flash column chromatography on silica gel with neat hexanes as an eluent. **CAUTION:** Mercaptans are pungent and toxic compounds with a relatively high volatility. Aromatic thiols are also more acidic than the homologous phenols.

#### 2.2.2 Synthesis of 1,1-Bis­(1′-naphthylthio)­methane (**L1**)

The synthesis was adapted from a literature protocol.^44^ To a 50 mL round-bottom flask was dissolved 1-naphthalene mercaptan (500 mg, 3.12 mmol) in anhydrous ethanol (3 mL). An excess of potassium hydroxide (210 mg, 3.74 mmol) was added, and the mixture was allowed to stir at room temperature for 30 min, whereupon dissolution of the potassium hydroxide was observed. A stoichiometric amount of diiodomethane (0.13 μL, 418 mg, 1.56 mmol) was added dropwise. **CAUTION:** Diiodomethane is a known carcinogen and is highly volatile. An exothermic reaction was observed, and the mixture was allowed to stir at room temperature for 1 h. A white precipitate of potassium iodide could be observed at the bottom of the flask, and the mixture was refluxed for 1 h to bring the reaction to completion. Upon cooling, the reaction mixture was cooled to room temperature, and a triphasic mixture was observed: white precipitate, yellow oil, and cloudy off-white supernatant. The desired product was extracted using a mixture of water and dichloromethane. The organic layers were combined and dried with anhydrous sodium sulfate. The solvent was removed using reduced pressure to yield the crude product as a yellow oil. This oil was subjected to flash column chromatography on silica gel using a mixture of ethyl acetate and hexane (1:9) as an eluent. The pure product was isolated as a clear yellowish oil (472 mg, 91%). ^1^H NMR (400 MHz, CDCl_3_) δ (ppm): 8.34 (m, 2H), 7.87 (m, 2H), 7.81 (m, 2H), 7.74 (m, 2H), 7.52 (m, 4H), 7.42 (m, 2H), 4.45 (s, 2H). ^13^C NMR (100 MHz, CDCl_3_) δ (ppm): 134.0, 133.3, 131.9, 130.9, 128.6, 128.5, 126.6, 126.2, 125.5, 125.1, 41.3. FTIR ν̅ (cm^–1^): 3056 (w), 2921 (w), 1562 (m), 1501 (m), 1458 (w), 1408 (w), 1381 (m), 1333 (m), 1261 (m), 1187 (m), 1146 (w), 1119 (w), 1067 (w), 1024 (m), 973 (m), 949 (m), 846 (m), 784 (s), 766 (s), 728 (m), 662 (s), 538 (s), 520 (m), 472 (m), 438 (m), 404 (m), 355 (m).

#### 2.2.3 Synthesis of **CP1**

In a vial were added copper­(I) iodide (115 mg, 604 μmol) and acetonitrile (2 mL). To this suspension was slowly added a solution of **L1** (100 mg, 301 μmol) in acetonitrile (3 mL). Taking care not to shake the mixture, the reaction was left undisturbed for 1–4 h to yield large off-white to yellowish block crystals suitable for SCXRD. For further analyses, the crystals were scraped from the walls of the vial and rinsed (but not soaked) in acetonitrile. The identity of the product was confirmed by PXRD. No change in the PXRD pattern was observed after mechanical crushing. The compound yellows after prolonged UV irradiation; this color change is not observed under visible light. *T*_f_ > 200 °C (dec.) *T*_dec_ = 289 °C (Figure S1). Anal. Calcd. for **CP1**: C, 35.4; H, 2.23; S, 8.99. Found: C, 34.4; H, 2.30; S, 8.53. FTIR ν̅ (cm^–1^): 3054 (w), 2907 (w), 1590 (w), 1569 (w), 1502 (w), 1375 (w), 1357 (w), 1336 (w), 1254 (w), 1135 (w), 1062 (w), 1024 (w), 967 (w), 814 (m), 787 (s), 760 (s), 721 (s), 664 (m), 643 (m), 619 (m), 531 (m), 514 (m), 473 (w), 468 (w), 420 (m), 404 (m), 377 (w).

### 2.3 Characterization Methods

#### 2.3.1 Photophysical Characterization

The UV–vis spectra in solution were acquired with an HP 8452A diode array spectrometer using 1 × 1 cm^2^ quartz cuvettes. The UV–vis spectra in the solid state were acquired on a Varian Cary 300 Bio UV–vis spectrometer equipped with an integration sphere using grazing angle reflectance. Samples were prepared by dispersing the powder between two quartz plates. The luminescence spectra in solution or the solid state were acquired on either an Edinburgh Instruments FLS980 or a Horiba PTI QTM-400. Both spectrofluorometers were equipped with Xe lamps and single–singlet monochromators. Solution samples were prepared in quartz cuvettes (room temperature) or NMR tubes (liquid nitrogen temperature). Solid-state samples were prepared in a sealed borosilicate glass capillary. All spectra were corrected for the instrument response. The fluorescence lifetime measurements for **L1** were performed on an Edinburgh Instruments FLS980 using a microchannel plate PMT and nano-LED lasers (fwhm = 120 ps). The measurements were performed by using the TCSPC method, and the data were treated by multiexponential deconvolution analysis with the minimum number of components required to reach the best fits. The phosphorescence lifetime measurements of **L1** and **CP1** were performed on an Edinburgh Instruments FLS980 using a microchannel plate PMT and Xe-flash arc lamp (fwhm = 50–100 μs depending on instrumental setup). The measurements were performed by using the gated emission method, and the data were treated by multiexponential deconvolution analysis with the minimum number of components required to reach the best fits.

#### 2.3.2 Crystallographic Methods

A single crystal of **CP1** was mounted on a Bruker D8 Venture four-circle diffractometer equipped with an Oxford Cryosystems nitrogen jet stream low-temperature system. X-ray radiation was generated with Mo Kα radiation (λ = 0.71073 Å). The radiation was monochromated through graphite from a microfocus IμS tube from Incoatec GmbH. Applying a least-squares fit to the optimized setting angles of the entire set of collected reflections yielded the lattice parameters. Intensity data were recorded as ϕ and ω scans with κ offsets. No significant intensity decay or temperature drift was observed during data collection. SAINT version 8.73A software was used to reduce the data, and absorption correction was performed using SADABS-2016/2. Structure elucidation was performed by using SHELXT with intrinsic phasing. The H atoms were placed geometrically and refined on a riding model. Full-matrix least-squares on *F* ^2^ was carried out using the SHELXL program on the complete set of reflections. All non-hydrogen atoms were refined with anisotropic displacement parameters, whereas H atoms were treated in a riding mode.^59–61^

Powder X-ray diffraction patterns were acquired from a small sample of powder dispersed over a ZERO diffraction plate from Charles Supper Company and placed for analysis on a Bruker D8 Advance diffractometer. Diffractograms were acquired using DIFFRAC.COMMANDER (version 8.6.3.0) software from Bruker using Cu-Kα (λ = 1.54060 Å) as an X-ray source (40.0 kV, 40.0 mA). The data were collected from 4 to 60° (2θ) over 2046 steps (0.0274°·step^–1^; 0.50 s·step^–1^) using a LynxEye detector. No further data treatment or baseline was performed on the acquired diffractograms.

#### 2.3.3 Other Characterization Methods

FTIR spectra were recorded on an Agilent Cary 630 FTIR spectrometer using an ATR module with a resolution of 0.931 cm^–1^. Nuclear magnetic resonance (NMR) spectra were recorded on a Bruker Advance AS300 or AS400 NMR spectrometer. Chemical shifts are given in parts per million relative to the residual solvent peak. Phase transitions were sought out through differential scanning calorimetry using a DSC Q200 instrument from TA Instruments. Three scans were made between −10 and 200 °C (10 °C min^–1^) under the nitrogen atmosphere, with the upper bound being determined from the visual decomposition of the **CP1** material determined by melting point analysis. No phase transition was observed. Thermal stability was assessed by calculating the onset temperature of degradation from a thermogravimetric analysis curve acquired by using a ThermoFisher thermal analyzer. The heating rate was set at 25 K min^–1^ under nitrogen atmosphere starting at room temperature, and finishing at 1073 K. Elemental analysis (C, H, S, O, N) measurements were acquired in triplicate by using a Flash 2000 OEA from Thermo Fisher Scientific. The limit of detection for nitrogen, oxygen, and sulfur is 0.05%.

### 2.4 Computational Methods

#### 2.4.1 Molecular DFT Modelization

##### 2.4.1.1 General Methods

All molecular DFT computations were run using Gaussian 16 as a software.^62^ In order to accurately model excited states, all computations used the PBE0 functional and LANL2DZ basis set to accurately represent the heavy atoms in the system. Simulated UV–vis spectra were obtained through purely singlet TD-DFT computations to model the lowest-lying excited states and associated oscillator strengths. The data was extracted and interpreted using GaussSum 3.0.^63^ The molecular orbitals were rendered at an isovalue of 0.02. The red lobes are positive, while the green lobes are negative. No polarization functions or solvent models were employed.

##### 2.4.1.2 Ground State Calculations

A computational fragment of **CP1** consisting of a single cubane cluster and its surrounding ligands (Cu_4_I_4_ **L1**_4_) was trimmed from the solved *.cif file. The coordinates were slightly adjusted (loose symmetry criterion) to fit C_2_ point symmetry. This motif was optimized to its minimum energy conformation under C_2_ point group symmetry constraints. To elucidate the excimer behavior of **L1**, a single ligand molecule was trimmed from **CP1**’s solved *.cif file and used as is. The “ground state” of the excimer was obtained by running a single-point energy calculation on this fragment, while the true minimum energy conformation was obtained by a geometry optimization.

##### 2.4.1.3 Excited State Optimizations

The lowest-lying triplet excited states of **CP1**, **L1**, and **L1** (excimer) were obtained by optimizing the triplet geometry (unrestricted DFT) of each fragment using the guess = read keyword. For **CP1**, this optimization was constrained to the C_2_ point symmetry. For **CP1**, an attempt was made to optimize both the ^3^M/XLCT and ^3^CC states by selectively populating the LUMO or LUMO+8, respectively, as indicated by literature methods.^64^ However, both computations led to the ^3^M/XLCT state, suggesting that the ^3^CC state either does not exist or rapidly converts to the ^3^M/XLCT state through internal conversion (see results and discussion below).

##### 2.4.1.4 Phosphorescence Wavelength Calculations

The optimized triplet excited state geometries were then subjected to mixed singlet–triplet TD-DFT computations by using the electronic configuration of the ground state as the initial state (restricted DFT). The lowest-lying triplet excitation corresponding to the HOMO–LUMO transition was found from the *.log file and used as an approximation for the phosphorescence wavelength.

#### 2.4.2 Plane-Wave DFT Modelization

The electronic structure calculations for the bulk **CP1** solid were conducted using plane-wave density functional theory (PW-DFT) supported by Quantum Espresso 7.3.1. as a software.^65–67^ All computations were run using the standard solid-state pseudopotentials (SSSP-efficiency version 1.3.0.),^68^ with the PBE functional. A crude electronic wave function was obtained at the Γ *k*-point through a self-consistent field calculation. This step was refined to a sampling of eight *k*-points in the first Brillouin zone through a nonself-consistent field calculation to yield the Fermi energy and bandgap. The band structure was obtained by extending the previously obtained results to a k-path terminated by the seeK-path algorithm,^69^ which was then adjusted to accurately sample the first Brillouin zone at critical points. Molecular orbital renderings were obtained at the Γ *k*-point with Quantum Espresso 7.3.1. and visualized using VESTA freeware.^70^ Yellow lobes are positive, and blue lobes are negative.

## 3 Results and Discussion

### 3.1 Synthesis and X-ray Diffraction

Copper­(I) iodide was reacted with **L1** as ligand upon letting the ligand-containing solution flow slowly over the CuI-containing solution, where microcrystals appeared. The material was identified as coordination polymer **CP1**, 1D-[Cu_4_(μ_3_-I)_4_(μ_2_-**L1**)_2_]_*n*_, whose structure was elucidated through single crystal X-ray diffraction (SCXRD, Figure 1 and Table S1). The structure is built with closed cubane Cu_4_I_4_ nodes exhibiting short intercopper distances (2.72 Å average), which is often a sign of cuprophilic interactions (the van der Waals radius of copper is 1.40 Å). This distance is practically always a sign of a low-energy optically active ^3^CC state.^42^ The Cu_4_I_4_ clusters are bridged to each other by two **L1** ligands through Cu–S coordination bonds, forming Cu_2_(μ_2_-I)_2_(μ_2_-**L1**)_2_Cu_2_(μ_2_-I)_2_ loops. It is noteworthy that these macrorings experience some stress as evidenced by the short center-to-center distance between the two cubane units (7.81 Å). The overall 1D-chain packing is also accompanied by a synergistic stacking of naphthyl fragments through π-stacking, adopting a slightly slipped dimer geometry (3.7 Å interplanar distance).

**Figure 1:**
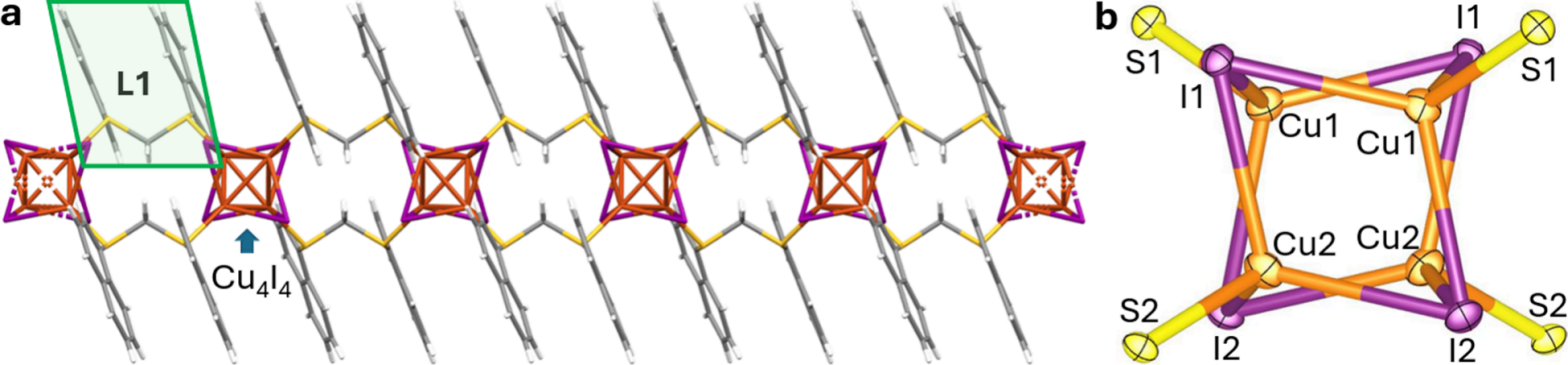
(a) Structural representation of a segment of CP1 obtained using Mercury. Orange: copper, violet: iodine, yellow: sulfur, gray: carbon, white: hydrogen. A single L1 fragment is highlighted in green. (b) Structural representation of the Cu_4_I_4_S_4_ node. Bond distances: Cu1–Cu1 = 2.6739(7) Å; Cu1–Cu2 = 2.8275(6) Å; Cu2–Cu2 = 2.6188(11) Å; Cu1–I1 = 2.6248(7) Å; Cu2–I1 = 2.7722(6) Å; Cu1–I2 = 2.7515(5) Å; Cu2–I2 = 2.6312(6) Å; Cu1–S1 = 2.3157(7) Å; Cu2–S2 = 2.3064(9) Å.

The structural prediction regarding copper­(I) halide coordination polymers is notoriously difficult, with few apparent patterns.^42^ However, as mentioned above, obtaining a 1D-CP exhibiting cubane nodes is not unprecedented, and rather common when using similar ArSCH_2_SAr ligands.^42,44,56,57^

The phase purity of **CP1** powder was verified through good agreement between the powder X-ray diffraction pattern with the simulated one generated from the single crystal diffraction data (Figure S2).

Furthermore, no phase change was observed when heating the sample (by DSC), except for a thermal degradation above 200 °C, witnessed by a color change. Upon changing the number of CuI and **L1** equivalents in the reaction, no M:L ratio dependence was observed as **CP1** was the only product formed. This outcome is to be expected for the S-(CH_2_)_1_-S chain due to high ligand strain.^44,56,57^ However, this is not necessarily the case for all chain lengths as several CuI/ArS­(CH_2_)_*m*_SAr mixtures involve a molar M:L ratio dependence on the resulting motif of the (CuI)_*m*_ SBU (globular versus quasiplanar) and CP dimensionality (1D-, 2D-, 3D-).^42^ This synthesis phenomenon is more frequent with longer −(CH_2_)_*m*_– central flexible chain (*m* ≫ 1).^43,44^

### 3.2 Ligand Photophysical Properties

#### 3.2.1 Singlet Excited States

**L1** is a new molecule and its photophysical properties were assessed prior to addressing those for **CP1**, which contains both luminophores (1-naphthyl pendant group and Cu_4_I_4_ cluster).^40,41^ Indeed, the data will allow for comparison and assignment with those of **CP1** to help understand how these units interact, if this is indeed the case. Naphthalene, as the simplest poly arene, is known to be luminescent when excited with a UV-A radiation.^50–52^ It is known to be fluorescent and form excimers,^71,72^ and to be phosphorescent, also forming triplet excimers when subjected to the heavy atom effect.^73–75^

The steady-state spectra and time-resolved luminescence spectra of **L1** in solution have been acquired (Figure 2a and Table 1). **L1** exhibits fluorescence at 450 nm (Figure 2a). Again, it is noteworthy that naphthalene is not as well-known for excimer formation in comparison with other polyarenes, such as the extensively documented pyrene and derivatives.^76^ Since two naphthyl residues are near each other in **L1**, excimer formation becomes more likely than that in single naphthalenes in solution. Compounded with the fact that this band is found in the same region as other cases of excimers of di­(naphthalene) and that it is a large and featureless band even at cryogenic temperatures (Figure S4), the emission behavior of this band is attributed to an “excimer” configuration that does not upconvert back to the “monomer” spectral signature.

**Figure 2:**
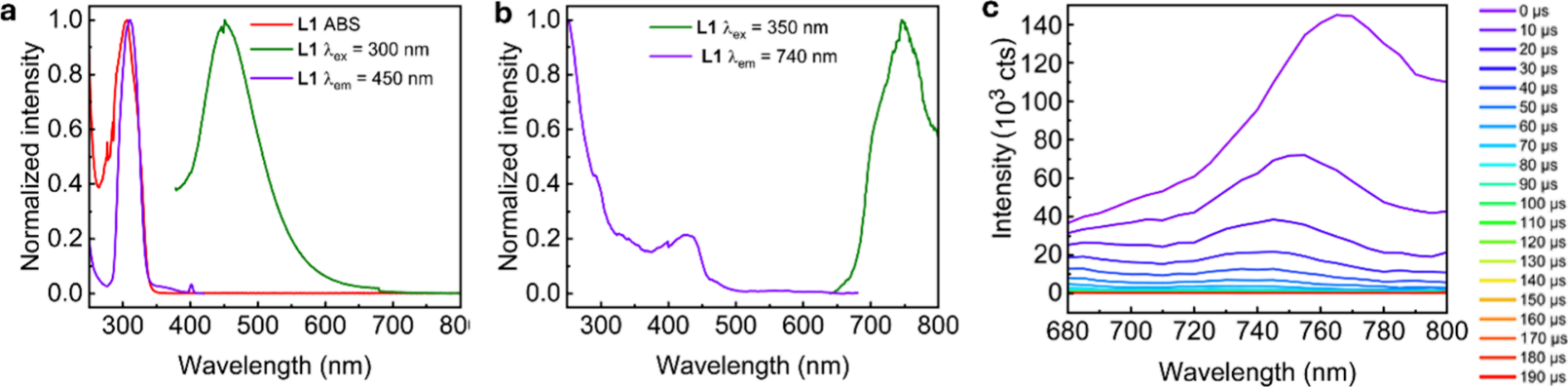
(a) Steady-state absorption (red), emission (green), and excitation (purple) spectra of **L1** in 2-MeTHF at 293 K. (b) Steady-state emission (green) and excitation (purple) spectra of **L1** in n-PrBr solution at 77 K. (c) Time-resolved emission spectra of **L1** in n-PrBr solution at 77 K (the delay times are placed on the right-hand side). The isolated bands exhibit maxima at 740 and 765 nm.

**Table 1:** Photophysical Parameters for **L1** in 2-MeTHF Solution^a^

parameters	293 K	77 K
λ_abs_ (nm)	306	n.d.^b^ ^,^ ^c^ ^,^ ^d^
ε (M^–1^cm^–1^)	7047	
λ_em,excimer_ (nm)	450	442
τ_PL,excimer_ ^e^ *f_i_* (%)^f^	66 ps (5.23%)^g^,^h^	560 ps (11.2%)
	1.0 ns (26.4%)	1.6 ns (29.6%)
	3.9 ns (16.4%)	3.6 ns (41.2%)
	9.0 ns (51.9%)	8.8 ns (18.0%)

a The supporting spectra and photoluminescence decay curves are found in Figures S2–S4.

b n.d.: no data.

c n.a.: not applicable, as this band is not observed.

d n.d.: no data, as the emission band is higher in energy than the most energetic TCSPC laser.

e The lifetime components were extracted through an exponential fit of the decay data using the model $I(t)=\sum_{i=1}^{n}B_{i}\exp(-t/\tau_{i})$ , where each component’s relative error is ± 10%.

f Percent contributions are of the form $f_{i}(\%)=B_{i}\tau_{i}/\sum_{j=1}^{n}B_{j}\tau_{j}$ .

g This component is smaller than the instrument response function (IRF, ∼150 ps) and cannot be assigned with confidence.

h The observation of multiple lifetime contributions is attributed to the fact that **L1** exhibits two S–C_naphthyl_ and two S–CH_2_ bonds, leading to many possible configurations, two of which present preorganized face-to-face bis­(naphthyl) assemblies in a *syn* or *anti* form, in addition to conformers where the naphthyl groups are far from each other. All these conformers are in equilibrium with each other with different barriers of activation to interconversion, exhibiting in their own emission quantum yields, with their own temperature-dependency on their quantum yields.

#### 3.2.2 Triplet Excited States

In preparation for its inclusion in the heavy-atom-rich **CP1**, **L1**’s phosphorescence properties are of high interest. However, the relatively light S atoms did not induce a sufficiently fast intersystem crossing to efficiently populate the T_1_ state. Free naphthalene molecules are known to form singlet and triplet excimers.^53–55^ However, the phosphorescence of naphthalene excimer is poorly reported.^50–52,77^ To the best of our knowledge, no example of triplet emission arising from linked dinaphthyl as well as their excimer emission has been reported.

Measurements in bromopropane solution were therefore conducted to take advantage of the external heavy atom effect (i.e., increase of spin–orbit coupling and rate of intersystem crossing) to increase the population of the triplet states. A phosphorescence spectrum was recorded for **L1** (Figure 2b), and time-resolved emission spectroscopy (TREmS) permitted one to depict two structureless bands decaying at different rates (Figure 2c). The excitation spectrum exhibits a feature in the 400–500 nm region (purple line; a band and a shoulder) tentatively assigned to singlet–triplet absorptions (enhanced via a heavy atom effect). Signals in this similar range have previously been reported for standalone naphthalene.^55^ The photoluminescence lifetimes (τ_PL_’s) of the two bands (740 and 765 nm), and two emission time scales were investigated: μs- and ms/s-scale (Table 2). Based on the findings on the singlet excited states’ study above, the 740 and 765 nm bands are respectively assigned to a triplet “monomer” excited state and a triplet excimer state.

**Table 2:** Photophysical Parameters of **L1** Phosphorescence in n-BrPr Solution at 77 K^a^

parameters	^3^monomer	^3^excimer
λ_em_ (nm)	740	765
τ_PL_ ^b^ *f_i_* (%)^c^	2.9 μs (10.5%)^d^	
	9.0 μs (69.6%)	3.4 ms (20.8%)
	20 μs (20.0%)	38 ms (79.2%)
χ^2^	0.992	1.070

a Supporting photoluminescence decay curves are found in Figures S5 and S6.

b The lifetime components were extracted through an exponential fit of the decay data using the model $I(t)=\sum_{i=1}^{n}B_{i}\exp(-t/\tau_{i})$ , where each component’s relative error is ± 10%.

c Percent contributions are of the form $f_{i}(\%)=B_{i}\tau_{i}/\sum_{j=1}^{n}B_{j}\tau_{j}$ .

d Multiple triplet lifetimes are depicted, which can be attributed to the fact that, upon cooling to 77 K, not only aggregation of **L1** could occur but also n-PrBr forms microcrystals. Then, **L1** may occupy different sites in and on these microcrystals, thus providing different environments.

#### 3.2.3 Computational Investigations

To solidify these assignments, we performed DFT and TD-DFT calculations. Two conformers were designed to model the excimer and “monomer” excited states (Figure 3a). A preliminary TD-DFT study allowed for the simulation of singlet–singlet absorption spectra (Figure 3b). The similarity between the experimental absorption spectrum and the simulated spectra is not particularly striking, which may be due to the presence of a mixture of both conformers in solution. These conformers may rapidly interchange through a thermally allowed activation. Nevertheless, based on DFT data (details placed in the SI), both conformers exhibit ππ* low-energy singlet excited states.

**Figure 3:**
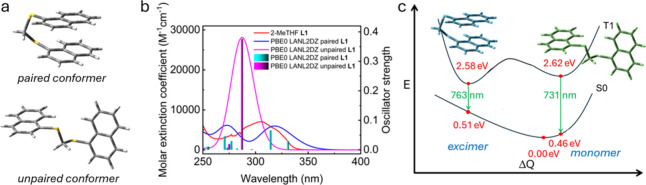
(a) Conformers used for computations: paired conformer simulates the excimer; unpaired conformer simulates the “monomer”. The atomic coordinates are given in Tables S2 and S3. (b) Simulated UV–vis spectra for two different conformations of L1, compared to experimental spectrum (red; solvent = 2-MeTHF, 293 K). The bars represent the individual electronic transitions, to which an arbitrary full width at half-maximum (fwhm) of 3000 cm^–1^ was added to obtain the full blue and fuschia line simulated spectra. A table of the transitions used to model this spectrum and relevant molecular orbitals is available in Tables S4, S5, Figures S7, and S8. (c) Proposed schematic potential curves of the S_0_ and T_1_ states of the conformers (not to scale).

The HOMO → LUMO transition was selectively excited in the triplet regime to assess the optimized geometries and total energies of the monomer and excimer in the triplet excited states. The resulting geometries are rather similar to those of the ground state ones (Tables S6 and S7). Running single-point energy (DFT) and TD-DFT calculations on the conformers using the ground state wave function allows for the extraction of their excitation energies (Table 3 and Figure 3c).

**Table 3:** Predicted Phosphorescence Parameters for **L1** ^a^

prediction	monomer	excimer
ΔSCF_DFT_ (eV)^b^	2.16	2.07
TD-DFT λ_P_ (nm)	731	763
experimental λ_P_ (nm)	740	765
difference (cm^–1^)	–166	–34
relative difference	–1.23%	–0.260%

a A list of electronic transitions, and molecular orbitals are available in Tables S8, S9, Figures S9, and S10.

b ΔSCF_DFT_: difference in DFT self-consistent field energies.

### 3.3 Photophysical Properties of CP1

#### 3.3.1 Steady State Spectra of CP1

The closed cubane Cu_4_I_4_S_4_-containing CPs are renowned for being systematically strongly emissive from their low-energy ^3^CC state at room temperature, based on reported emission quantum yields.^42^ Surprisingly, this is not the case for **CP1** as it is optically silent at room temperature, even under laser excitation. Concurrently, only faint luminescence is noted at 77 K (Figure 4a). Moreover, the excitation spectra of the emission maxima matched only parts of the absorbance spectrum. The excitation–emission map discriminated in fact three different emission bands (Figure 4a–c and Table 4), thus indicating the presence of two anti-Kasha emissions. At first glance, such a behavior is indicative that 2 or 3 luminophores emit in a noninteractive way. This situation may appear not unusual for Cu_4_I_4_ cubane-containing materials, where high-energy ^3^M/XLCT and low-energy ^3^CC emissions are often observed.^42,78^ As an example, the classic 0D-Cu_4_I_4_Py_4_ emits through these states with varying relative intensities depending on temperature.^42,64,78^ The emission quantum yield for cubane-based CPs is also generally quite high (Φ_PL_ > 0.2).^44^ However, none of these observations were the case for **CP1** as it is nonemissive.

**Figure 4:**
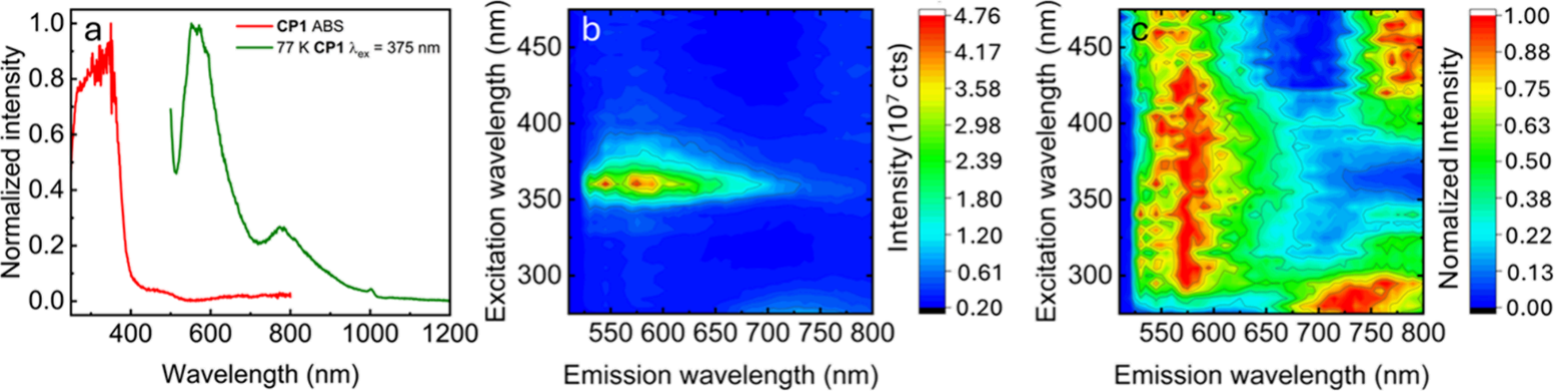
(a) Steady-state absorption (red) and emission (green) spectra of CP1 at 77 K. (b) Raw excitation–emission map of CP1 at 77 K. (c) Excitation–emission map in which each component has been normalized, providing evidence for different bands.

**Table 4:** Wavelength Maxima of the Absorption and Emission Bands for CP1 at 77 K

band label	wavelength maxima
λ_abs_	324 and 460 nm (weak)
A λ_em_	573 nm (λ_ex_ = 360 nm)
B λ_em_	732 nm (λ_ex_ = 235 nm)
C λ_em_	785 nm (λ_ex_ = 454 nm)

The **CP1** case differs as the emission bands are red-shifted. Indeed, the literature places the high-energy band maxima in the 400–500 nm range (573 nm for **CP1**), and the low-energy band maxima in the 550–600 nm window (732–785 nm for **CP1**).^42,43,64^ While the difference may stem from the nature of the aromatic group attached to the heteroatom ligand (generally non- and substituted phenyl or pyridine, compared to 1-naphthyl in **CP1**), other phenomena are found to be responsible.

#### 3.3.2 Time-Dependent Spectroscopic Measurements

The time-dependent spectroscopic measurements were performed for these three bands (Table 5).

**Table 5:** Emission Decay Profiles for CP1 at 77 K^a^

Measurement	A (573 nm)	B,C^b^ (732 nm, 785 nm)
τ_PL_ ^c^ (*f_i_*)^d^ short components	n.d.^e^	4.4 μs (11.1%)^f^
		13 μs (21.1%)^f^
		450 μs (67.7%)
τ_PL_ (*f*_i_) long components	3.0 μs (56.7%)^f^	
	160 μs (0.867%)	670 μs (42.9%)
	1.8 ms (4.43%)	4.8 ms (57.1%)
	7.1 ms (38.0%)	

a The associated photoluminescence decay curves are available in Figures S11–S13.

b Due to their close proximity, these bands could not be resolved.

c The lifetime components were extracted through an exponential fit of the decay data using the model $I(t)=\sum_{i=1}^{n}B_{i}\exp(-t/\tau_{i})$ , where each component’s relative error is ±10%.

d Percent contributions are of the form $f_{i}(\%)=B_{i}\tau_{i}/\sum_{j=1}^{n}B_{j}\tau_{j}$ .

e Not determined: as these components were too weak and too short to yield a good fit.

f This component is smaller than the IRF (∼50–100 μs), and cannot be determined with confidence.

The combination of the low emission intensity (wider slits) and the proximity of emission maxima renders the emission decays’ intertwinement. As a result, similar emission lifetimes are depicted in the multiphasic decays. To help the interpretation, time-resolved emission and excitation spectra (TREmS; TRExS, respectively) were conducted (Figure 5), and three observations can be made. First, the average decay components for bands **A** and **C** are longer than those for the **B** band. This is due to the emission spectra at longer delay times, which show a major intensity arising from band **A**, along with some minor contributions from bands **B** and/or **C**. Second, an examination of the TRExS indicates that the excitation spectra at similar delay times exhibit excitation bands associated with bands **A** and **C** (Figure 4). Finally, the excitation and emission bands for **B** are found to be mainly associated with relatively short delay times and thus shorter lifetimes. These observations thus provide a clearer interpretation of the lifetimes and assignments (Table 6).

**Figure 5:**
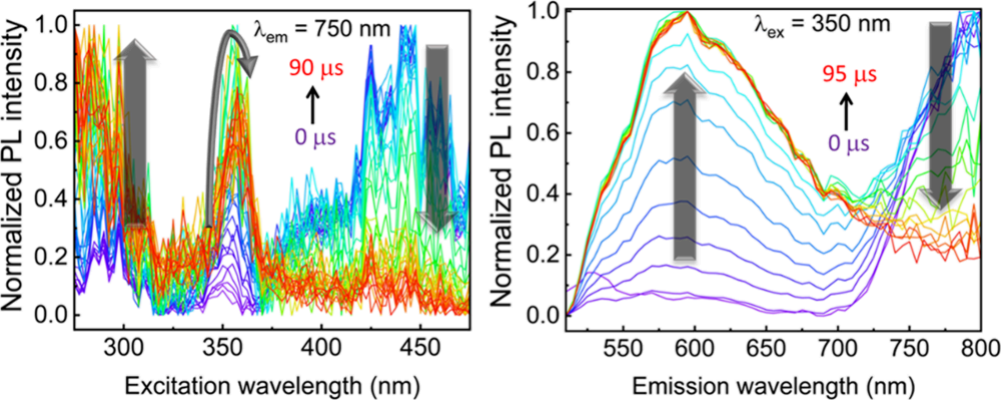
TRExS and TREmS of CP1 at 77K. Left: excitation spectra, λ_em_ = 750 nm; 2 μs separates each line. Right: emission spectra, λ_ex_ = 350 nm; 5 μs separates each line. Absolute intensity spectra and the normalized TRExS of the band at 600 nm are shown in Figures S14–S17.

**Table 6:** Assignment of Approximate Lifetime Contributions for Each Emission Band

band labels	λ_P_	τ_PL_
A and C	573 and 785 nm	∼1, ∼6 ms
B	732 nm	<50–100 μs, ∼450 μs

At first glance, the presence of two distinct time scales, μs (band **B**) and ms (bands **A** and **C**), suggests ^3^CC and ^3^ M/XLCT excited states, respectively.^78^ It is noteworthy that the triplet emission lifetimes of naphthalene as a guest molecule inside 4-dimethylamino-pyridine and poly­(methyl methacrylate) matrices at 293 K are 1.79 and 0.5 s, respectively.^79^ However, ligand phosphorescence, often decaying in the μs-time scale, can also occur as reported for other (CuI)_*n*_-containing CPs.^41^ The lower values listed in Tables 4 and 5 are due to the heavy atom effect. Indeed, the phosphorescence lifetimes of *N*-naphthyl methylamine (NMA) of the perovskite (PEA)_1.4_(NMA)_0.6_PbBr_4_ (PEA = 2-phenylethylamine cation) is 3.02 ms (618 nm).^80^

#### 3.3.3 DFT and TD-DFT Computations

Adopting the same approach used by Ford and co-workers on the 0D-cluster Cu_4_I_4_Py_4_, geometry optimizations of a candidate ^3^MXCLT state and a candidate ^3^CC state were performed (Experimental Section).^64^ The *.cif file was truncated to a single Cu_4_I_4_ **L1**_4_ fragment and subjected to geometry optimization under the *C*_2_ point group. This symmetry constraint is necessary to obtain pure wave functions while optimizing the excited states.^64^ The list of atomic coordinates for this optimized *C*_2_-Cu_4_I_4_ **L1**_4_ fragment is shown in Table S10. The computed HOMO → LUMO+*n* transitions (*n* = 0–7) give rise to the formation of the spin-allowed lowest-energy M/XLCT states with dominant 1-naphthyl π-contributions (Figure 6). Concurrently, HOMO → LUMO+8 generates a CC state built with Cu_4_I_4_ atomic contributions with obvious bonding Cu···Cu interactions (Figure 6).

**Figure 6:**
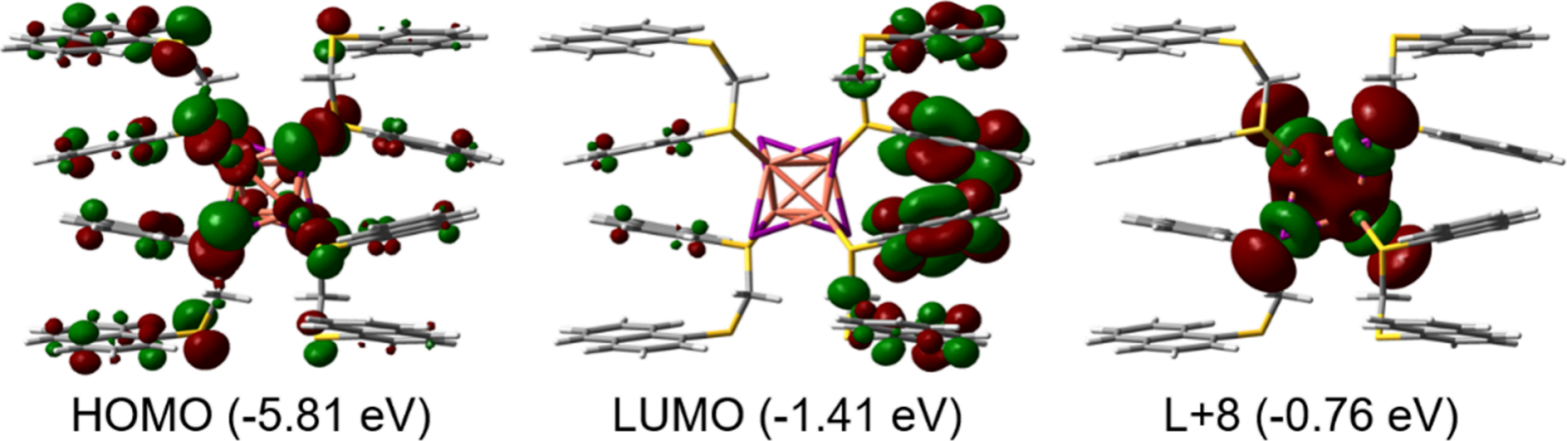
Representation of relevant frontier MOs in the Cu_4_I_4_L1_4_ fragment.

Upon selective excitation of the HOMO → LUMO transition, the geometry optimization of the lowest energy triplet excited state, the ^3^ M/XLCT state, was computed. The atomic contributions of the LSOMO and HSOMO confirm the CT nature of this excited state with Cu–Cu separations too long (average 2.76 Å) to imply a ^3^CC excited state (Figure S18).^42,64^ However, upon selective excitation of the HOMO → LUMO+8 transition, the optimized geometry and nature of the resulting LSOMO and HSOMO are the same as those for the ^3^ M/XLCT state. The atomic coordinates of this ^3^ M/XLCT-Cu_4_I_4_ **L1**_4_ fragment are available in Table S11.

The fact that the anticipated ^3^CC state is not observed as the lowest-lying excited state of a closed cubane-Cu_4_I_4_ cluster, thus contrasting the literature, is intriguing, and hypotheses are put forward.^42,78^ First, it may be possible that such a state exists at a lower energy level but converts nonradiatively to the ground state instead of emitting due to the high overlap between the potential curves this would cause. Second, it may be possible for the ^3^CC state to exist either at a higher or lower energy relative to the ^3^M/XLCT state, but it rapidly converts nonradiatively to the latter due to a higher potential curve overlap.

Nevertheless, this ^3^M/XLCT fragment was subjected to single-point energy (DFT) and TD-DFT calculations using the wave function of the ground state, yielding relative energies and a simulated phosphorescence wavelength (Table 7).

**Table 7:** Key Predictions Pertaining to Cu_4_I_4_L1_4_’s ^3^M/XLCT Phosphorescence^a^

prediction	Cu_4_I_4_L**1**_4_
ΔSCF_DFT_ (eV)	3.14
TD-DFT λ_P_ (nm)	574

a A list of the relevant transitions and molecular orbitals is available in Table S12 and Figure S19.

Another important question arising from the experimental data is the excitation-dependence of the observed emission bands (Figure 4). TD-DFT computations were performed to generate a simulated spectrum using the ground state fragment of Cu_4_I_4_ **L1**_4_ to address this point (Figure 7).

**Figure 7:**
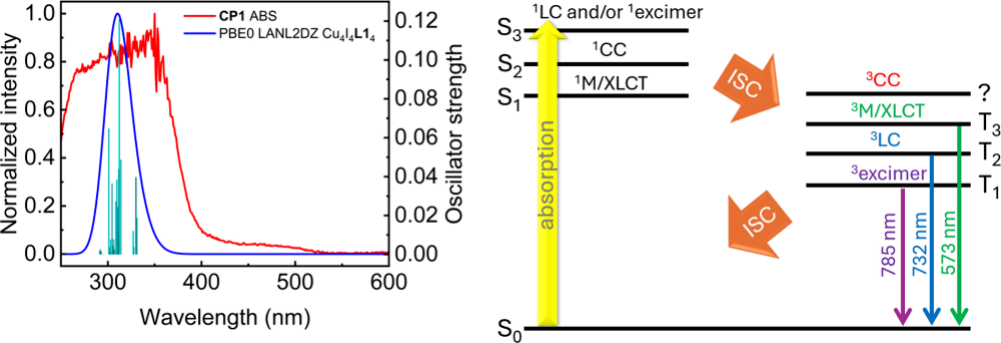
Left: Simulated (TD-DFT) UV–vis absorption spectra for Cu_4_I_4_L1_4_, compared to the experimental spectrum of CP1. The bars represent the individual spin-allowed (S_0_ → S_*n*_) electronic transitions, to which an arbitrary full width at half-maximum (fwhm) of 3000 cm^–1^ was added to obtain the full line spectrum (blue). A table of the calculated electronic transitions used to model this simulation and the relevant representations of the MOs are, respectively, placed in Table S13 and Figure S20. The absorption feature in the 400–550 nm range (red line) most likely contains S_0_ → T_*n*_ components. Right: energy state diagram summarizing the observed absorption and triplet state emissions, all of which are corroborated by computations in CP1. Note that the intersystem crossings, ISC, dominate the nonradiative processes.

The lowest-lying singlet–singlet transition corresponds to the excitation of the ^1^ M/XLCT state (n°1, 331 nm), as expected in similar compounds.^64^ On the other hand, the ^1^CC excitation is found higher in energy (n°17, 305 nm): this is also well-documented.^64^ Concerning the ^1^LC excitations, these could unfortunately not be located in these calculations due to severe issues involving disk memory. Nevertheless, from the experimental data (Figure 2, Figure 5), it can be reasonably hypothesized that these electronic transitions would occur at wavelengths slightly lower than those observed for the ^1^ M/XLCT states based on the spectroscopic features of the data of the standalone **L1** above. TD-DFT computations performed on the triplet excited states permitted us to locate a group of ^3^LC electronic transitions in the 490–496 nm region (Table S14). This computational outcome corroborates the presence of a weak feature in the 400–550 nm range (Figure 7 (left), red line) and confirms the population of the excited state leading to the presence of the **C** emission band (λ_em_ = 785 nm) when excited in the 420–475 nm region (Figure 4). All in all, singlet–triplet electronic transitions are spin-forbidden, and when the chromophores are in the presence of heavy atoms, such as iodide and copper, enhanced spin–orbit couplings render them less forbidden and more observable through augmented rates of intersystem crossing (ISC).^81,82^

#### 3.3.4 Plane-Wave DFT Simulations

The assessment of electronic communication between unit cells along given axes can be assessed through plane-wave-assisted DFT calculations. The computed band structure of **CP1** was obtained at critical points in the first Brillouin zone, which is provided as results in Figure 8. Note: the bandgap obtained with this method (2.00 eV) is generally very underestimated.^83–85^

**Figure 8:**
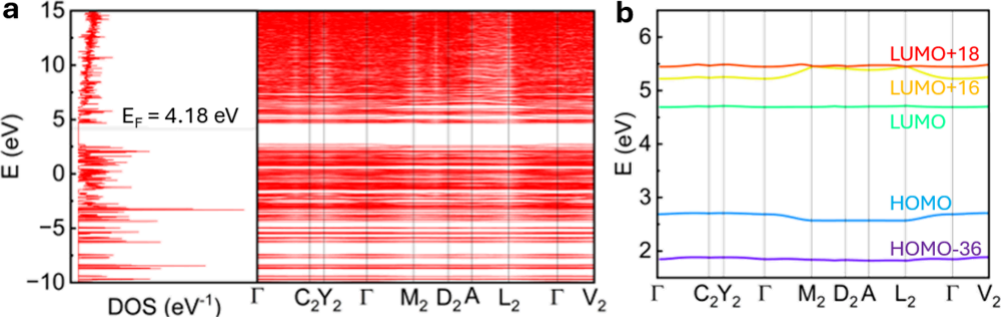
Left: density of states (DOS) diagram and band structure of CP1 obtained with the PBE functional and SSSP-efficiency pseudopotentials. The first Brillouin zone and k-points are depicted in Figure 21 of ref 69. Right: energy of selected bands depends on the k-path. The representation for such bands is found in Figure S21. The orbitals were chosen due to their representation of the different chromophore systems in CP1: H-36 is the HOMO of the naphthyl system; H is the HOMO of the Cu_4_I_4_ system; L is the LUMO of the monomer naphthyl system; L+16 is the unoccupied orbital with bonding contributions between copper centers; L+18 is the LUMO of the excimer naphthyl system.

Very little structure-dependence in MO energies is depicted in **CP1**, except for the M_2_, D_2_, A, and L_2_ *k*-points which are different from the rest. This is significant because these *k*-points are placed on the c* vector along the *z*-axis (i.e., polymer direction), indicating that only modest electronic communication along the whole structure occurs. Thus, these computations indicate that no unexpected contributions from the solid-state structure are likely to occur. This computational conclusion is consistent with the fact that excitation migration does not occur in quasi-isolated Cu_4_I_4_L_4_.^86,87^

#### 3.3.5 Recapitulation and Spectroscopic Assignments

**CP1** is a triply emissive excitation-dependent luminophore (Figure 4). A detailed analysis of the ligand and **CP1**’s properties, and assignments of these three emission bands are made (Table 8). From a kinetic standpoint, the overall low intensity and emission time scale (Table 5) indicate a strong heavy atom effect (Figure 7 (right)).

**Table 8:** Spectroscopic Data and Emission Band Assignments of CP1 and Comparisons of the Key Experimental and Simulated Triplet Excited State Parameters

	experimental		**ΔSCF_DFT_** ^a^	TD-DFT		
	λ_P_		λ_*P*_	λ_P_		
	*E*		*E*	E		
band	**ν̅**	assignment	**ν̅**	**ν̅**	difference	relative difference
A	573 nm	^3^M/XLCT	395 nm	574 nm	–30 cm^–1^	–0.172%
	2.16 eV		3.14 eV	2.16 eV		
	17,452 cm^–1^		25,316 cm^–1^	17,422 cm^–1^		
B	732 nm	^3^LC	574 nm	731 nm	19 cm^–1^	0.139%
	1.69 eV		2.16 eV	1.70 eV		
	13,661 cm^–1^		17,422 cm^–1^	13,680 cm^–1^		
C	785 nm	^3^excimer	599 nm	763 nm	367 cm^–1^	2.88%
	1.58 eV		2.07 eV	1.62 eV		
	12,739 cm^–1^		16,694 cm^–1^	13,106 cm^–1^		

a ΔSCF_DFT_: difference in DFT self-consistent field energies.

## 4 Conclusions

**CP1** turns out to be stimuli-responsive, where at room temperature the material is not emissive (“off”) and (weakly) luminescent at low temperatures (“on”). This study falls in line with the work reported by Patterson et al. on the 0D-cubane complex Cu_4_I_4_(tetrathiophene)_4_, which modestly emits orange at 250 K, and bright yellow at 100 K.^88^ This color change is accompanied by a change in the average Cu···Cu distance. Concurrently, the related 1D-CP [Cu_4_I_4_(PhSCH_2_SPh)_2_]_*n*_ also undergoes a phase change from *C*2/*c* (115 K) to *P*2_1_/*c* (195 K), which is accompanied by a change in topology (zigzag at warmer temperatures vs linear), but the emission maxima (515 nm) and lifetimes (τ = 1.0 μs at 77 K, and 1.2 μs at 298 K) remain practically constant.^57^ Therefore, by comparison, **CP1** exhibits a better “on–off” behavior upon a temperature change, and this occurs in the absence of a phase change, presumably due to the tightness of the compact naphthyl contacts. The absence of emission at room temperature precludes exploring other stimuli such as solvato-^89^ and mechanochromism.^90^ To remedy this situation, future rational designs include the use of CuBr salt, which produces quasi-systematically rhomboid-Cu_2_Br_2_ SBU-containing CPs with thioether ligands, and which are also known as being emissive at room temperature from ^3^M/XLCT states.^42,43^ Moreover, the use of longer alkane (CH_2_)_*m*_ chains, namely, *m* = 6–9, has a higher propensity to form cubane Cu_4_I_4_S_4_-based SBUs with the correct stoichiometry,^91^ and at the same time may preclude the formation of excimers, which in the case of **CP1** contribute to generating lower excited states promoting extra nonradiative deactivation pathways. Finally, this detective endeavor permitted us to shine light on why the closed cubane Cu4I4S4 motif is exceptionally not emissive while all other CPs containing the motif are.

## Supplementary Material



## Notes

Supplementary photophysical characterization results, detailed crystallographic data, and supplementary computational results (PDF)
