# Supplementary material for: Design of a Triple Emissive Cu4I4 Coordination Polymer through Ligand Engineering
Source: Inorg Chem. 2025 Dec 12;64(51):25311–22. doi: 10.1021/acs.inorgchem.5c04789 (PMC12754793; doi:10.1021/acs.inorgchem.5c04789)
Supplement: Supplementary file 1 [file ic5c04789_si_001.pdf]

Supplementary information

Design of a Triple Emissive Cu_4I_4 Coordination Polymer Through Ligand Engineering

Léo Boivin, Daniel Fortin, Pierre D. Harvey*

Département de chimie de l'Université de Sherbrooke QC J1K 2R1 Canada

Pierre D. Harvey : pierre.harvey@usherbrooke.ca

TABLE OF CONTENTS

Figure S1. Determining the temperature of decomposition for **CP1** by TGA. S2
Table S1. Crystallographic data for **CP1**. S3
Figure S2. PXRD pattern for varying M:L ratios for synthesizing **CP1**. S4
Figure S3. Photoluminescence spectra of **L1** in 2-MeTHF at 77 K. S5
Figure S4. Photoluminescence decay of **L1** in 2-MeTHF. S6
Figure S5. Photoluminescence decay of **L1** in 2-MeTHF at 77 K. S7
Figure S6. Photoluminescence decay (short) of **L1** in bromopropane at 77 K. S8
Figure S7. Photoluminescence decay (long) of **L1** in bromopropane at 77 K. S9
Table S2. Atomic coordinates of the paired **L1** conformer. S10
Table S3. Atomic coordinates of the unpaired **L1** conformer. S11
Table S4. List of the first 10 singlet-singlet electronic transitions in **L1** (paired). S12
Figure S8. Representation of the frontier molecular orbitals in the paired conformer. S12
Table S5. List of the first 10 singlet-singlet electronic transitions in **L1** (unpaired). S13
Figure S9. Representation of the frontier molecular orbitals in the unpaired conformer. S13
Table S6. Atomic coordinates of the paired **L1** conformer in its triplet excited state. S14
Table S7. Atomic coordinates of the unpaired **L1** conformer in its triplet excited state. S15
Table S8. List of the first 5 singlet-triplet transitions in the paired **L1** (triplet geom.). S16
Figure S10. Representations of the frontier molecular orbitals of the triplet **L1** paired. S16
Table S9. List of the 5 first singlet-triplet transitions in the unpaired **L1** (triplet geom.). S17
Figure S11. Representations of the frontier molecular orbitals of the triplet **L1** (unpaired) S17
Figure S12. Photoluminescence decay of **CP1** at 77 K (**B**/**C** band, long) S18
Figure S13. Photoluminescence decay of **CP1** at 77 K (**B**/**C** band, short) S19
Figure S14. Photoluminescence decay of **CP1** at 77 K (**A** band, long) S20
Figure S15. Absolute TREmS for **CP1** at 77 K. S21
Figure S16. Absolute TRExS for **CP1** at 77 K (**B**/**C** bands) S22
Figure S17. Normalized TRExS for **CP1** at 77 K (**A** band) S23
Figure S18. Absolute TRExS for **CP1** at 77 K (**A** band) S24
Table S10. Atomic coordinates for the optimized Cu_4I_4**L1**$_4$ conformer S25
Figure S19. Frontier molecular orbitals in the triplet excited state for Cu_4I_4**L1**$_4$ S29
Table S11. Atomic coordinates for the Cu_4I_4**L1**$_4$ fragment in its triplet excited state S30
Table S12. First S-T transitions in the Cu_4I_4**L1**$_4$ (S0 wavefunction, ^{3}M/XLCT geometry) S34
Figure S20. Representation of the frontier MOs of the Cu_4I_4**L1**$_4$ in ^{3}MXLCT geom. S34
Table S13. List of the first 30 singlet-singlet transition in the Cu_4I_4**L1**$_4$ (ground state) S35
Figure S21. Representation of relevant molecular orbitals in the Cu_4I_4**L1**$_4$ (ground state) S36
Table S14. List of the first 10 singlet-triplet transitions in Cu_4I_4**L1**$_4$ (ground state) S37
Figure S22. Representation of relevant solid-state molecular orbitals of **CP1** S38
Figure S23. Excitation-emission map of **L1** in bromopropane solution at 77 K. S39

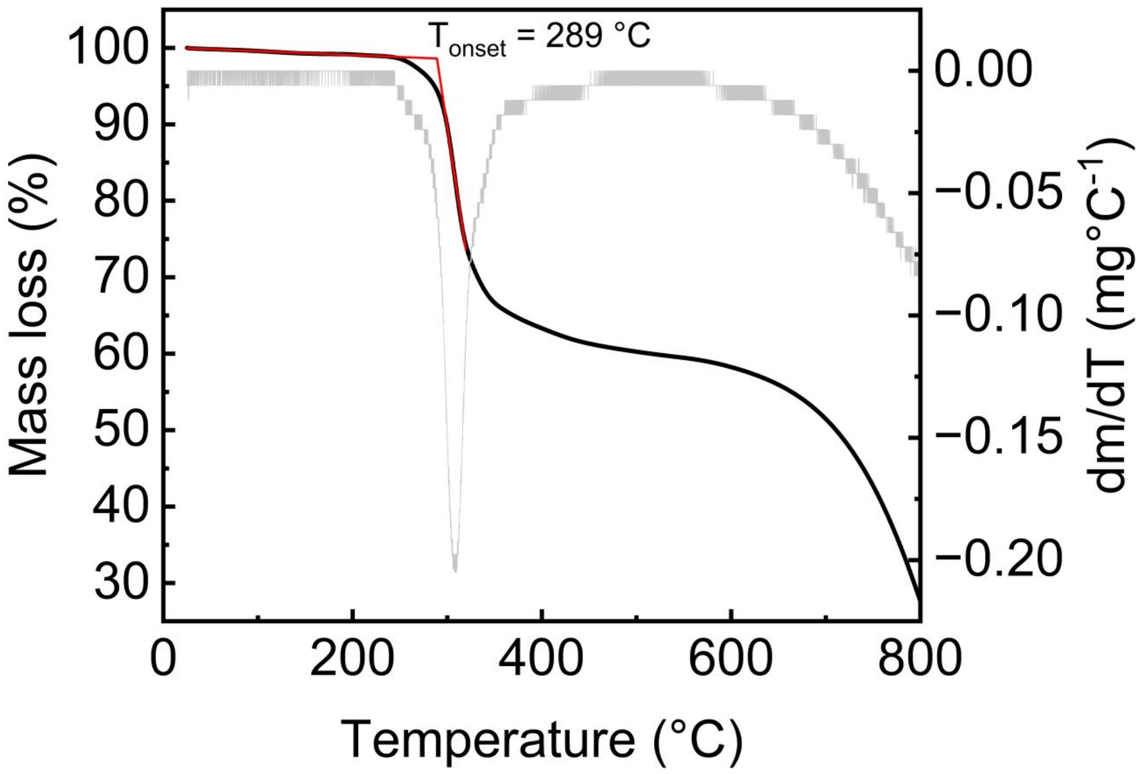

Figure S1. Determining the onset temperature of decomposition for **CP1** by thermogravimetric analysis (TGA).

Table S1. Crystallographic data for **CP1**.

Formula	$C_{21}H_{16}Cu_2I_2S_2$
Formula weight /g.mol^{-1}	713.34
Temperature /K	173(2)
Crystal system	Monoclinic
Space group	C2/c
a /Å	24.251(3)
b /Å	11.8891(14)
c /Å	15.5994(18)
α /°	90
β /°	111.3730(10)
γ /°	90
Volume /Å^3	4188.3(8)
Z	8
ρ_{calc} /gcm^{-3}	2.262
μ /mm^{-1}	5.183
F(000)	2704
Crystal size /mm^3	0.250 × 0.315 × 0.425
Radiation	Mo-Kα (λ = 0.71073 Å)
2θ range for data collection /°	2.19 to 26.43
Index ranges	$-27 \leq h \leq 30$<br>$-14 \leq k \leq 14$<br>$-19 \leq l \leq 19$
Reflections collected	28352
Independent reflections	4301 [R(int) = 0.0224]
Data/restraints/parameters	4301 / 0 / 244
Goodness-of-fit on F^2	1.202
Final R indexes [I ≥ 2σ (I)]	R1 = 0.0166, wR2 = 0.0373
Final R indexes [all data]	R1 = 0.0177, wR2 = 0.0376
Largest diff. peak/hole /eÅ^{-3}	0.385 and -0.489

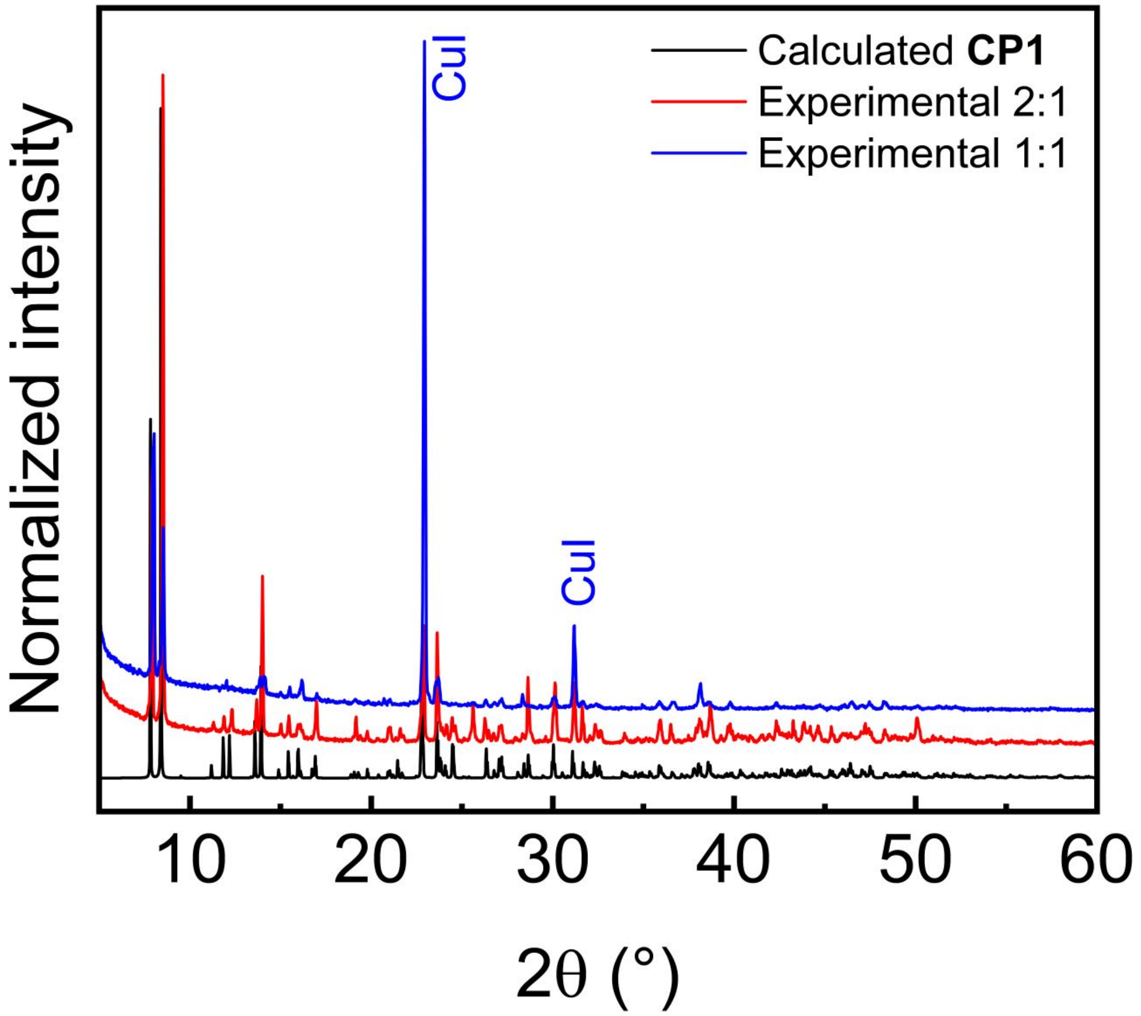

Figure S2. Powder X-ray diffraction patterns resulting from different M:**L1** ratio mixtures, compared to the simulated diffractogram from a single crystal X-ray data of **CP1**. CuI represents γ-CuI not reacted.

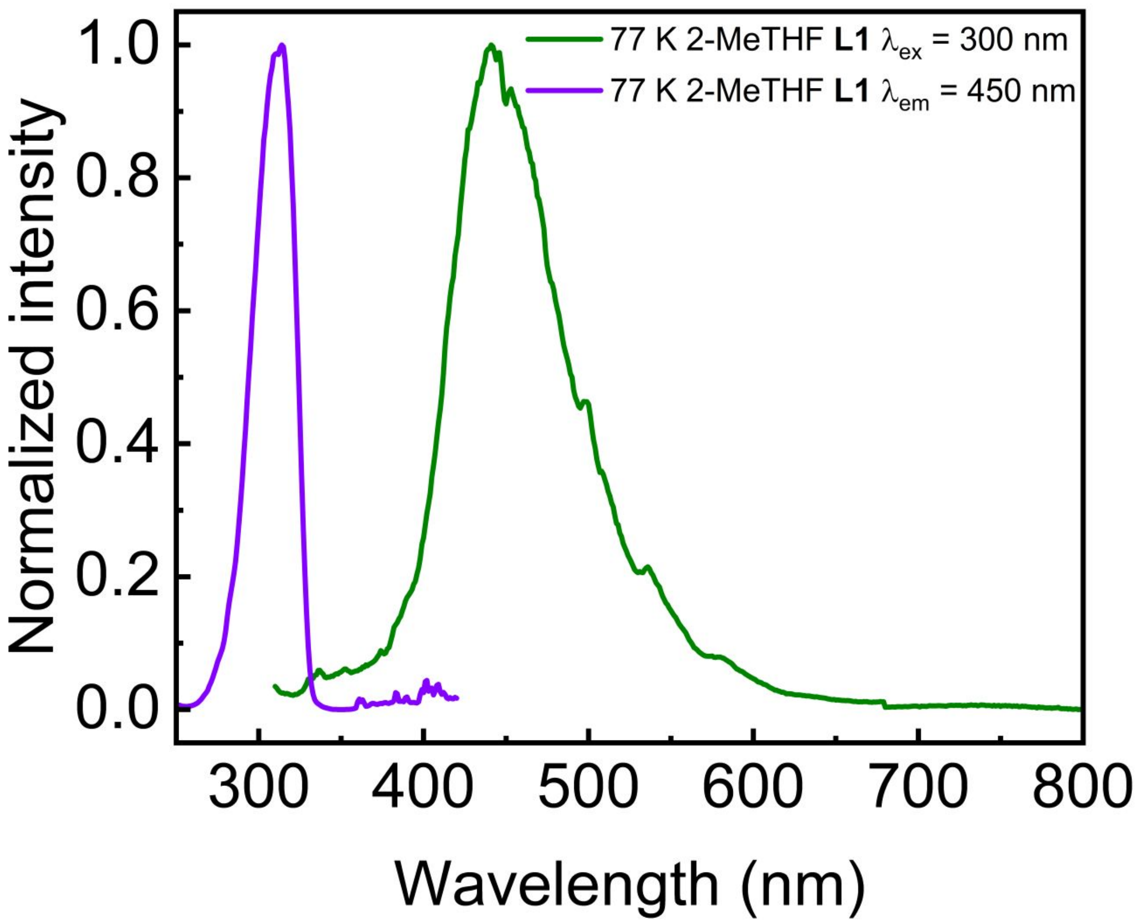

Figure S3. Photoluminescence (green) and excitation (purple) spectra of **L1** in 2-MeTHF solution at 77 K.

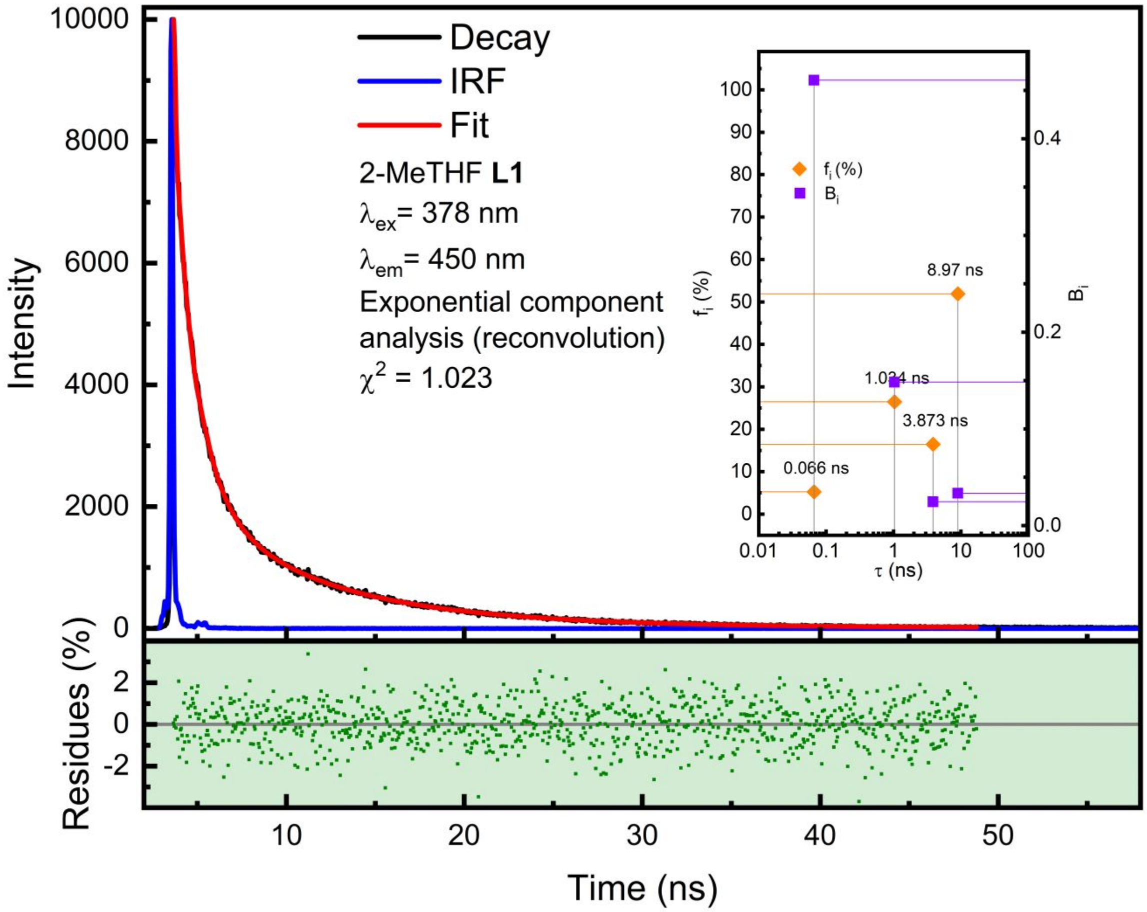

Figure S4. Photoluminescence decay curve of **L1** in 2-MeTHF.

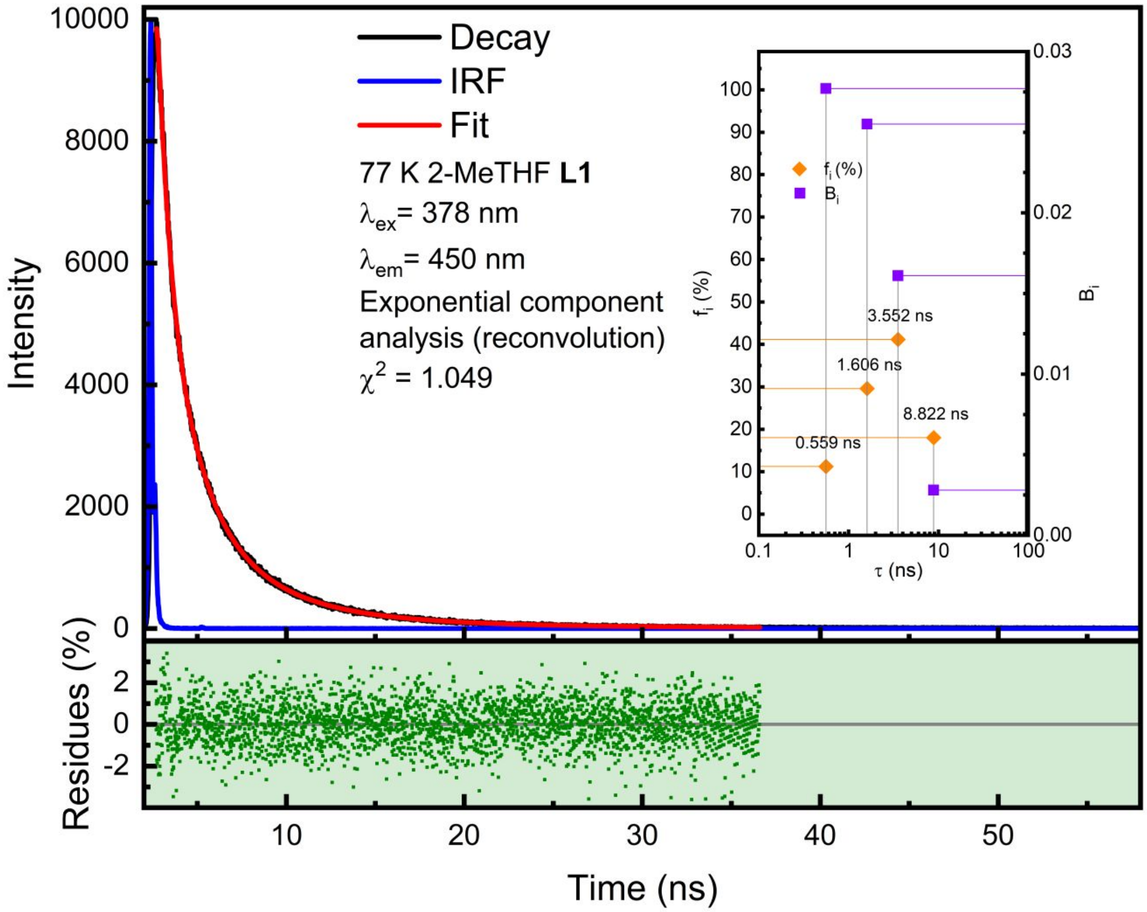

Figure S5. Photoluminescence decay curve of **L1** in 2-MeTHF solution at 77 K.

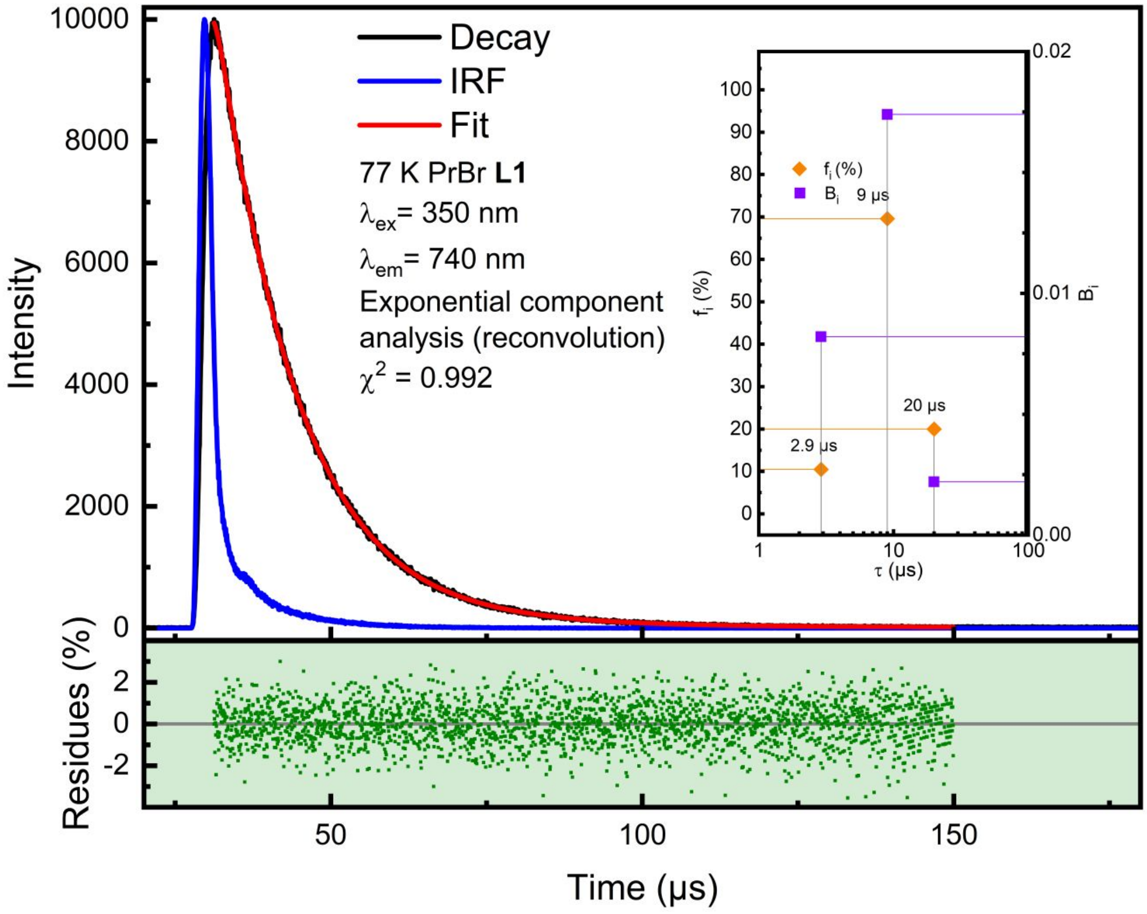

Figure S6. Photoluminescence decay curve (short components) of **L1** in bromopropane solution at liquid nitrogen temperature.

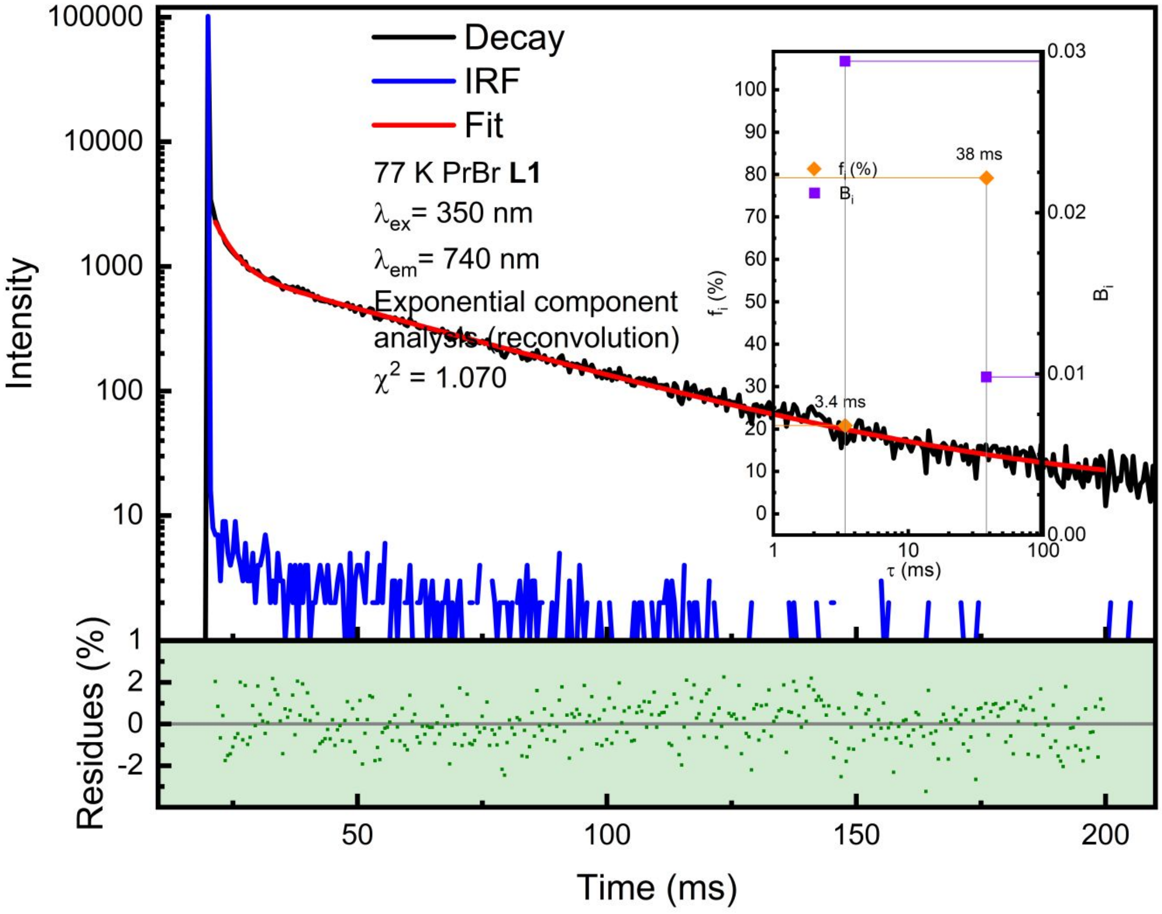

Figure S7. Photoluminescence decay curve (long components) of **L1** in bromopropane solution at liquid nitrogen temperature.

Table S2. Atomic coordinates of the paired conformer (see **Figure 3**).

C	-3.05026190	2.93376278	1.76720589
H	-3.36519092	2.27945997	2.43990749
H	-3.19203628	2.53017282	0.87410679
C	-4.27781543	4.65709875	3.66260486
C	-5.21145604	3.92531718	4.34590756
H	-5.75303298	3.29554687	3.88470947
C	-5.36872738	4.10937031	5.74290749
H	-6.00899620	3.59354122	6.21870936
C	-4.60305596	5.02542349	6.40750474
H	-4.71606324	5.13875697	7.34350472
C	-3.65149568	5.80204841	5.72620193
C	-3.46881095	5.63307933	4.31980194
C	-2.84298085	6.77853913	6.40149901
H	-2.95046037	6.90786303	7.33689896
C	-1.92997249	7.51874390	5.72739633
H	-1.40048474	8.15406229	6.19449443
C	-1.75920556	7.35391914	4.34349635
H	-1.11827536	7.88253742	3.88249445
C	-2.50898239	6.43744117	3.64919907
H	-2.38465060	6.33987968	2.71279905
C	-1.07070126	3.23459588	3.77870229
C	-1.79775555	2.43148011	4.61940479
H	-2.37966336	1.77122739	4.26200682
C	-1.67901963	2.58859009	6.01870434
H	-2.19618600	2.04224041	6.59940608
C	-0.82745174	3.51829142	6.54540143
H	-0.77512007	3.62694683	7.48730117
C	-0.02976121	4.31756363	5.70719883
C	-0.12472407	4.18337282	4.28879921
C	0.88519623	5.28037711	6.23389577
H	0.94905829	5.39529831	7.17479547
C	1.66050880	6.03199650	5.42069328
H	2.26245473	6.66453417	5.79549127
C	1.58475187	5.88321039	4.02249365
H	2.14138101	6.40774951	3.45899189
C	0.70698391	4.98315035	3.47089655
H	0.65789488	4.89613321	2.52579678
S	-4.09089481	4.42132858	1.89000497
S	-1.26985770	3.17524825	2.00250271

Table S3. Atomic positions of the unpaired conformer (see **Figure 3**).

C	0.00009292	0.00393291	-1.45057630
H	0.79311600	-0.40606099	-2.07789050
H	-0.79294456	0.41712195	-2.07577251
S	0.73373824	1.42921647	-0.46191418
S	-0.73349822	-1.42639235	-0.46917950
C	2.53063476	1.04553740	-0.58860036
C	3.15942832	0.08388669	0.26917060
C	3.27280083	1.76157382	-1.51413421
C	2.45263311	-0.67303222	1.24817028
C	4.57541543	-0.12748387	0.12744208
C	4.67280514	1.55189642	-1.63962892
H	2.77531495	2.49045647	-2.14600090
C	3.10997412	-1.59661075	2.04226122
H	1.38522261	-0.51320898	1.36480618
C	5.22238866	-1.08580997	0.96062907
C	5.30747048	0.62353153	-0.83696008
H	5.23419829	2.12514200	-2.37119615
C	4.50796646	-1.80876768	1.89837242
H	2.55392884	-2.16482567	2.78211070
H	6.29273062	-1.23916452	0.84577776
H	6.37754107	0.45466594	-0.93002888
H	5.00995770	-2.53766635	2.52798008
C	-2.53042902	-1.04227848	-0.59406932
C	-3.15948497	-0.08529749	0.26871938
C	-3.27236224	-1.75348669	-1.52350398
C	-2.45294900	0.66647717	1.25186130
C	-4.57547979	0.12663315	0.12790205
C	-4.67237665	-1.54333688	-1.64809014
H	-2.77468121	-2.47892308	-2.15917154
C	-3.11053853	1.58572131	2.05076110
H	-1.38553907	0.50616675	1.36782738
C	-5.22271223	1.08041868	0.96608283
C	-5.30728781	-0.61933752	-0.84059390
H	-5.23358163	-2.11275804	-2.38278181
C	-4.50853489	1.79846210	1.90777984
H	-2.55468671	2.15005152	2.79372270
H	-6.29305739	1.23423788	0.85188426
H	-6.37736739	-0.45012393	-0.93292438
H	-5.01072314	2.52392541	2.54118672

Table S4. List of the first 10 singlet-singlet electronic transitions in **L1** (paired conformer).

N°	λ (nm)	Osc. str.	Major contributions
1	331	0.0302	HOMO→LUMO (98%)
2	314	0.0661	HOMO→L+1 (95%)
3	282	0.0042	H-1→L+1 (16%), HOMO→L+2 (41%), HOMO→L+3 (12%)
4	277	0.0299	H-1→LUMO (32%), H-1→L+1(20%), HOMO→L+2 (25%)
5	275	0.0004	H-2→LUMO (79%)
6	273	0.0073	H-1→L+1 (19%), HOMO→L+3 (40%)
7	270	0.0460	H-1→LUMO (48%), H-1→L+1 (41%)
8	255	0.0104	H-4→LUMO (14%), H-2→L+1(11%), H-1→L+2 (12%), H-1→L+3 (16%), HOMO→L+2 (13%), HOMO→L+3 (18%)
9	254	0.0104	H-2→L+1 (76%)
10	249	0.0096	H-3→LUMO (74%)

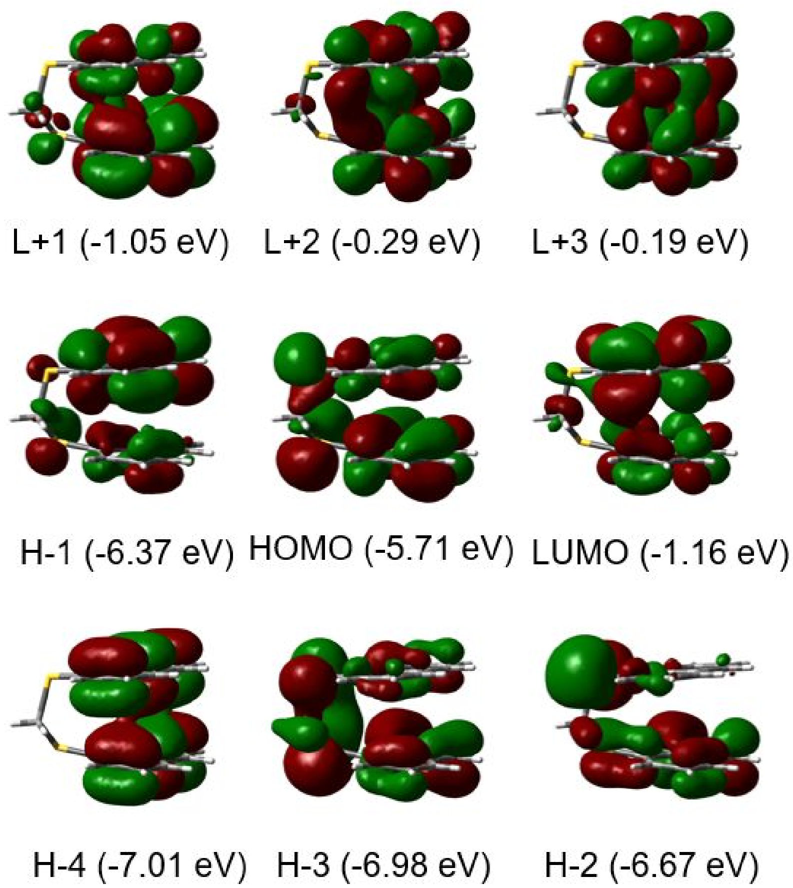

Figure S8. Representation of the frontier molecular orbitals in the paired **L1** conformer.

Table S5. List of the first 10 singlet-singlet electronic transitions in **L1** (unpaired conformer).

N°	λ (nm)	Osc. str.	Major contributions
1	296	0.0020	H-2→LUMO (46%), H-1→LUMO (23%), HOMO→L+1 (22%)
2	295	0.0002	H-2→L+1 (39%), H-1→L+1 (18%), HOMO→LUMO (34%)
3	287	0.3762	H-1→LUMO (38%), HOMO→L+1 (58%)
4	284	0.0005	H-2→L+1 (15%), H-1→L+1 (24%), HOMO→LUMO (58%)
5	275	0.0193	H-2→LUMO (43%), H-1→LUMO (30%), HOMO→L+1 (12%)
6	274	0.0000	H-5→LUMO (19%), H-4→L+1 (17%), H-2→L+1 (10%), H-1→L+1 (14%)
7	273	0.0016	H-5→L+1 (20%), H-4→LUMO (22%), H-1→L+2 (18%), HOMO→L+3 (22%)
8	271	0.0001	H-2→L+1 (32%), H-1→L+1 (38%)
9	254	0.0001	H-2→LUMO (89%)
10	250	0.0028	H-3→L+1 (90%)

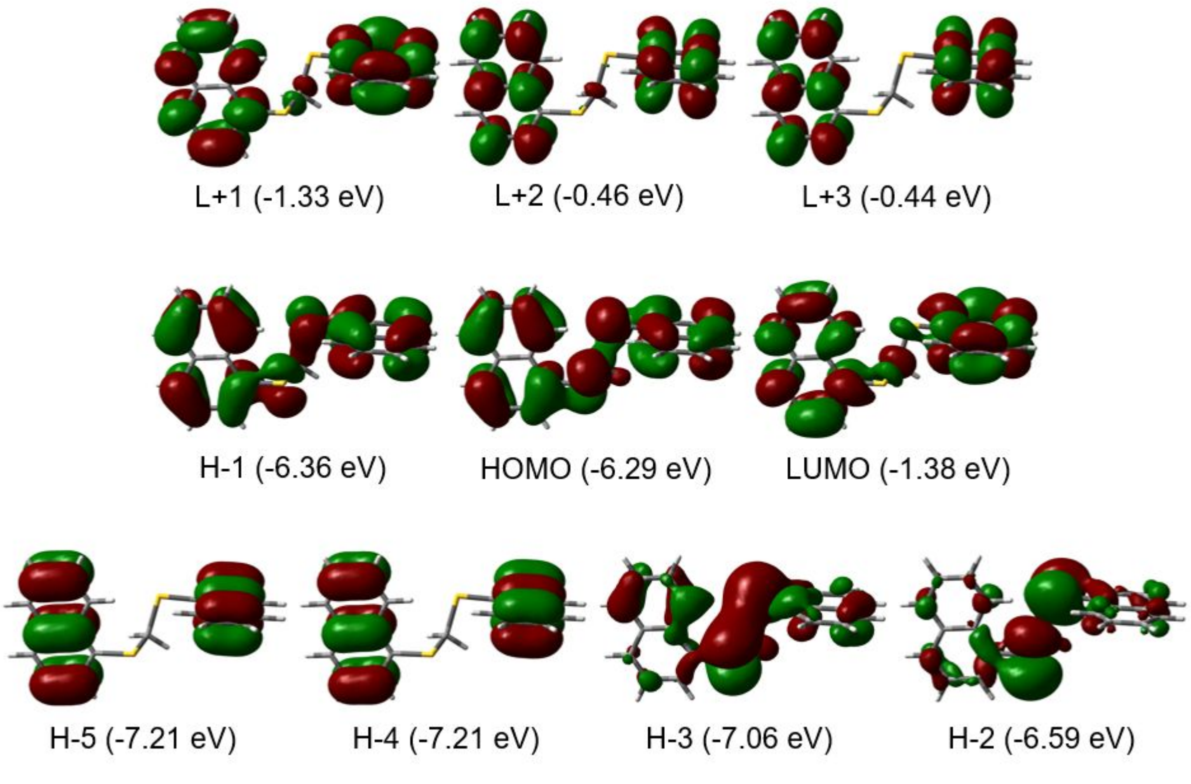

Figure S9. Representation of the frontier molecular orbitals in the unpaired **L1** conformer.

Table S6. Atomic coordinates of the paired **L1** conformer in its excited triplet state.

C	-3.17719639	0.75418238	-0.80284461
H	-3.34170591	1.05408633	0.23246076
H	-4.06927500	0.96889904	-1.39545624
C	-0.59382689	1.81581321	-0.19210115
C	-1.03157314	2.24904593	1.13456962
H	-2.07649547	2.49876832	1.29202785
C	-0.12311728	2.39012274	2.14937100
H	-0.44743620	2.72849031	3.12905013
C	1.26705330	2.10734407	1.92447268
H	1.98028351	2.21001350	2.73795335
C	1.73988338	1.71607782	0.63078001
C	0.79785717	1.56372977	-0.46060518
C	3.10323229	1.46774576	0.39553768
H	3.80692241	1.57792917	1.21741081
C	3.58024140	1.08219683	-0.89264098
H	4.64107950	0.90629679	-1.04402367
C	2.68252828	0.93589902	-1.93368128
H	3.02339494	0.64104435	-2.92172235
C	1.29001269	1.16535651	-1.71678054
H	0.59305953	1.01586407	-2.53569818
C	-1.61406228	-1.30767925	0.38230383
C	-1.90953583	-1.04106288	1.71285531
H	-2.90861383	-0.71510687	1.98776366
C	-0.92603530	-1.20804825	2.71828486
H	-1.17535755	-0.98260189	3.75075787
C	0.33652676	-1.66445643	2.38361283
H	1.09435571	-1.80098511	3.15108111
C	0.66278521	-1.97990580	1.03340373
C	-0.32238272	-1.79956523	0.00073888
C	1.95149432	-2.47541154	0.68525590
H	2.69020930	-2.60411492	1.47270607
C	2.26290034	-2.79140143	-0.62557818
H	3.24810742	-3.17192411	-0.87886896
C	1.29251913	-2.61080006	-1.64515367
H	1.54278590	-2.85441508	-2.67381554
C	0.03255035	-2.12071239	-1.34034951
H	-0.70425879	-1.97597033	-2.12389406
S	-1.79687789	1.78877719	-1.53887581
S	-2.93488363	-1.10047084	-0.87652669

Table S7. Atomic coordinates of the unpaired **L1** conformer in its triplet excited state.

C	0.15177900	-0.55042143	-1.22079693
H	1.08405297	-1.06212341	-1.46502539
H	-0.43837761	-0.38070468	-2.12226489
S	0.60128704	1.16656041	-0.54474170
S	-0.81700596	-1.67867157	-0.08824622
C	2.40910002	1.11644003	-0.48596370
C	3.14703295	0.08856380	0.19618230
C	3.10115751	2.19319578	-1.19474641
C	2.54908029	-0.88190793	1.01984916
C	4.58966799	0.06421384	0.03001122
C	4.46162610	2.16066299	-1.32833856
H	2.50819759	2.99046402	-1.63142599
C	3.32537992	-1.91725729	1.64012347
H	1.48264396	-0.83004953	1.22298614
C	5.32923882	-0.95771959	0.65582901
C	5.22556039	1.08585571	-0.73836511
H	4.97814694	2.94396894	-1.87477931
C	4.69208617	-1.95796002	1.45742675
H	2.82302657	-2.66101775	2.25106733
H	6.40855202	-0.98168353	0.52666612
H	6.30706487	1.07208295	-0.84342134
H	5.29123168	-2.73701276	1.91921320
C	-2.53587538	-1.11807284	-0.44555057
C	-3.16550792	-0.09434395	0.33585707
C	-3.22093139	-1.75489116	-1.46775927
C	-2.51750988	0.58658367	1.40731275
C	-4.52230288	0.26170819	0.01568895
C	-4.55993657	-1.39385693	-1.77666294
H	-2.72908721	-2.54221897	-2.03061937
C	-3.17600670	1.56955800	2.12486443
H	-1.49385892	0.32140977	1.65154684
C	-5.17204679	1.27871871	0.77391575
C	-5.19396747	-0.40501397	-1.04897628
H	-5.07750161	-1.90410555	-2.58326591
C	-4.51612413	1.92158868	1.80750991
H	-2.66561188	2.07873821	2.93703631
H	-6.19724593	1.54122713	0.52433621
H	-6.21916758	-0.12462480	-1.27819189
H	-5.01956323	2.69556278	2.37948267

Table S8. List of the 5 first singlet-triplet transitions in the paired **L1** conformer in its triplet excited state geometry. Since the computations did not include spin-orbit coupling, all the oscillator strengths are nil.

N°	λ (nm)	Osc. str.	Major contributions
1	763	0.0000	HOMO→LUMO (77%)
2	525	0.0000	H-1→LUMO (29%), H-1→L+1 (19%)
3	376	0.0000	H-2→LUMO (33%) HOMO→L+3 (14%)
4	345	0.0000	H-3→LUMO (17%)
5	337	0.0000	H-1→LUMO (13%), HOMO→L+1 (32%), HOMO→L+2 (15%)

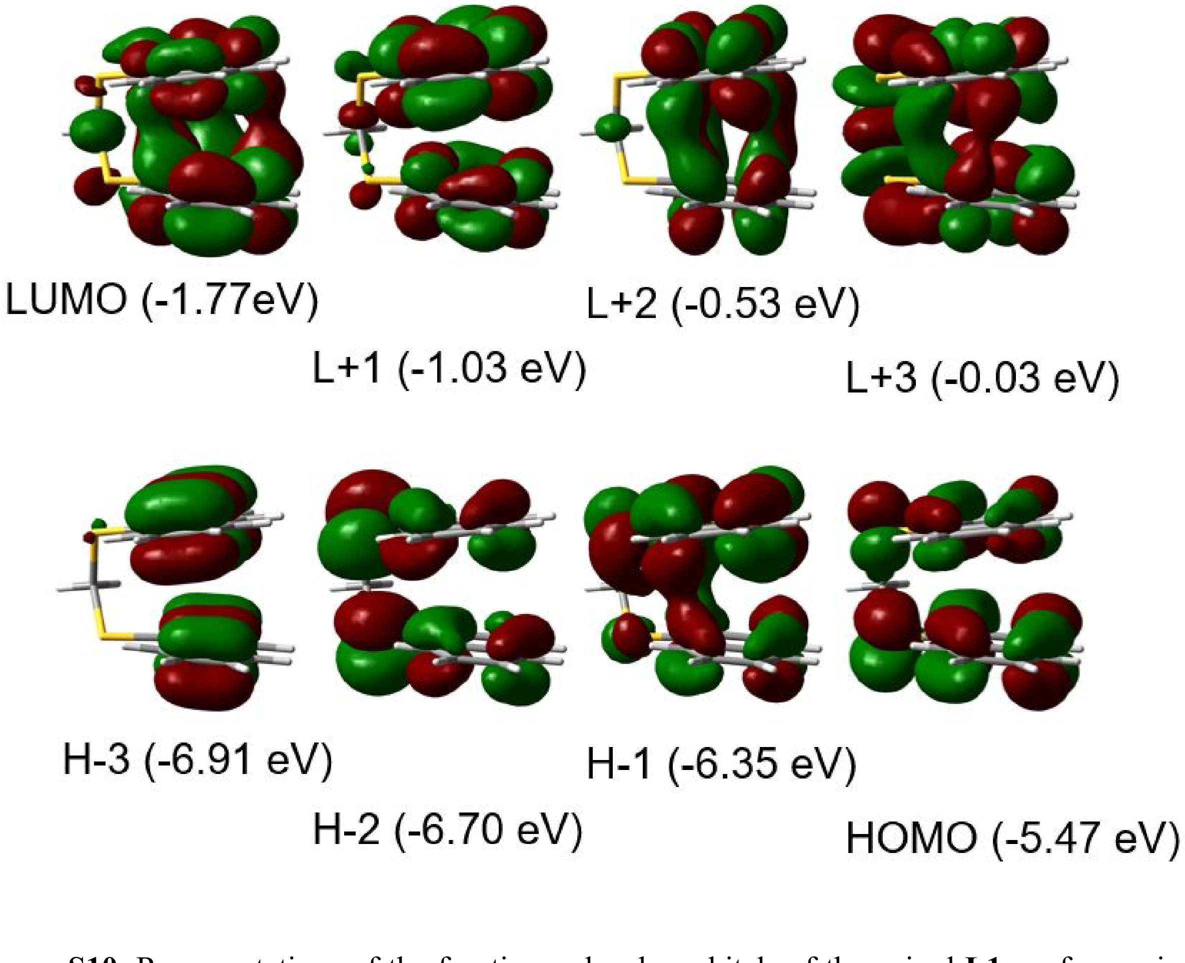

Figure S10. Representations of the frontier molecular orbitals of the paired **L1** conformer in its triplet excited state geometry.

Table S9. List of the 5 first singlet-triplet transitions in the unpaired **L1** conformer in its triplet excited state geometry. Since the computations did not include spin-orbit coupling, all the oscillator strengths are nil.

N°	λ (nm)	Osc. str.	Major contributions
1	731	0.0000	HOMO→LUMO (92%)
2	485	0.0000	H-1→L+1 (78%)
3	361	0.0000	H-3→LUMO (26%), H-2→LUMO (13%), HOMO→L+4 (14%), HOMO→L+5 (10%)
4	326	0.0000	H-5→LUMO (37%), HOMO→L+3 (54%)
5	322	0.0000	HOMO→L+1 (88%)

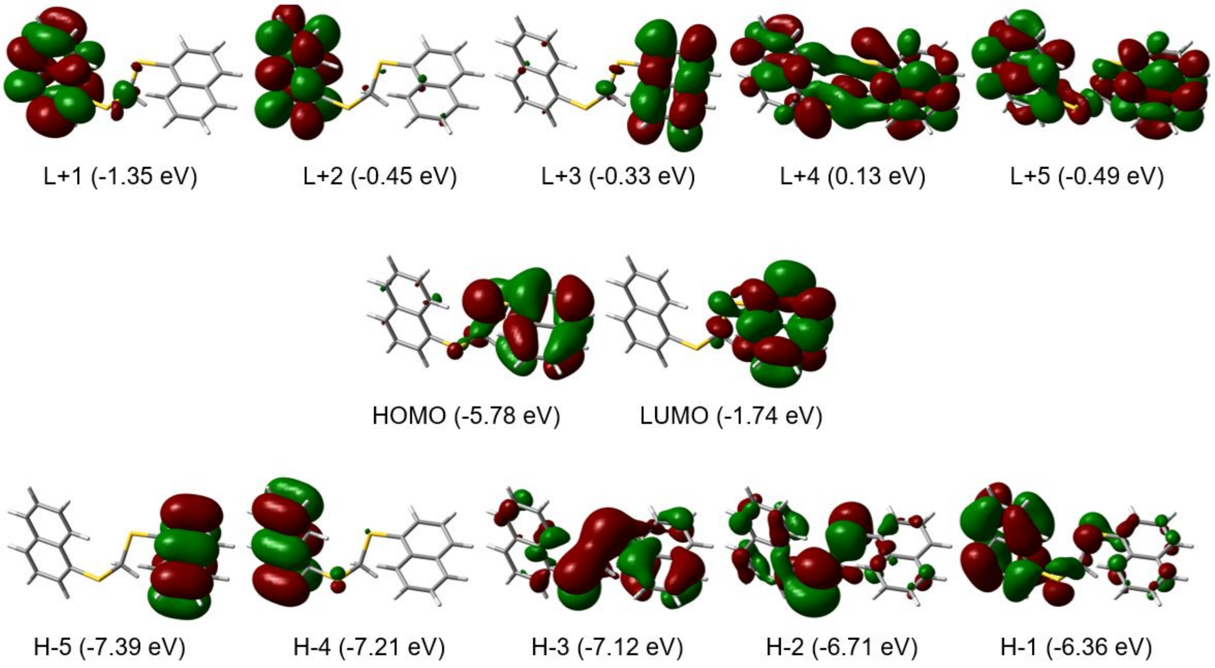

Figure S11. Representations of the frontier molecular orbitals of the unpaired **L1** conformer in its triplet excited state geometry.

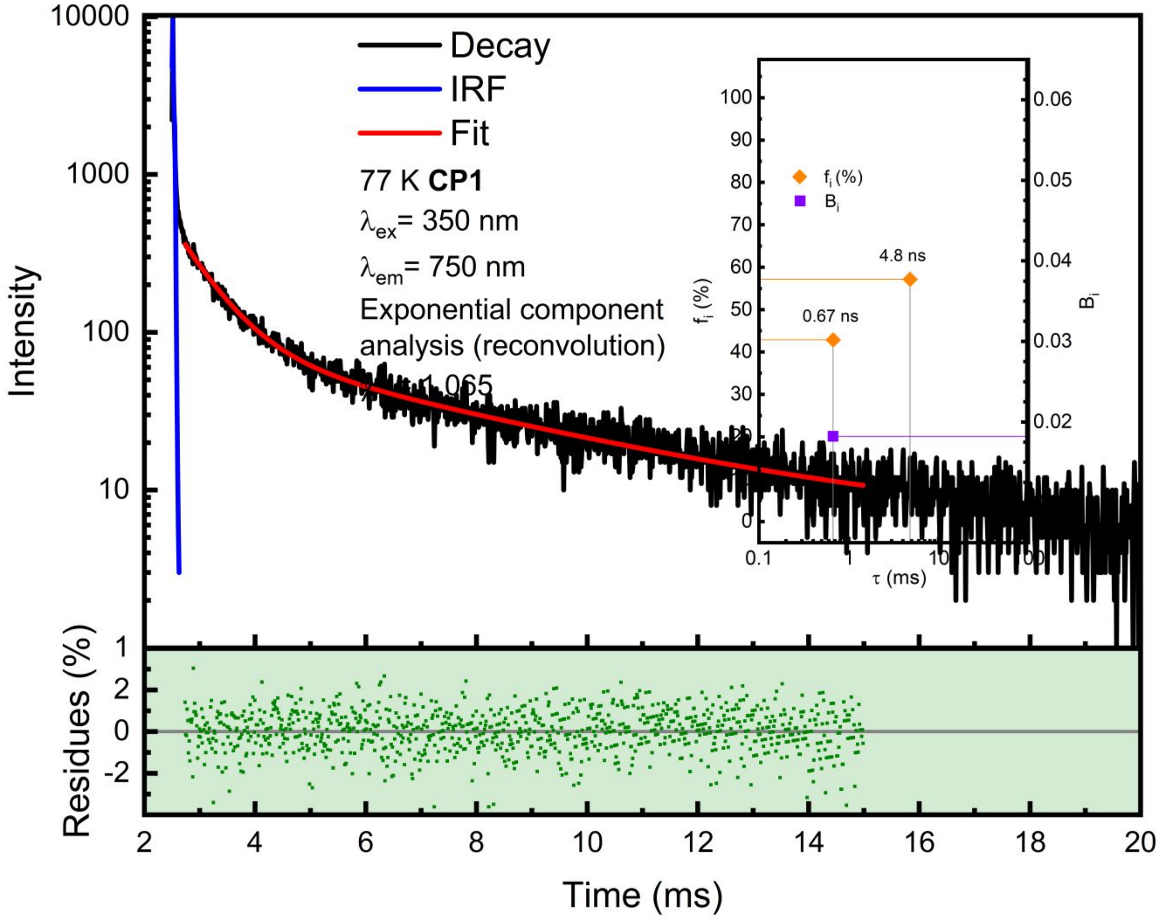

Figure S12. Photoluminescence decay curve of **CP1** at liquid nitrogen temperature. Depicted is the longer part of the decay of the **B/C** band.

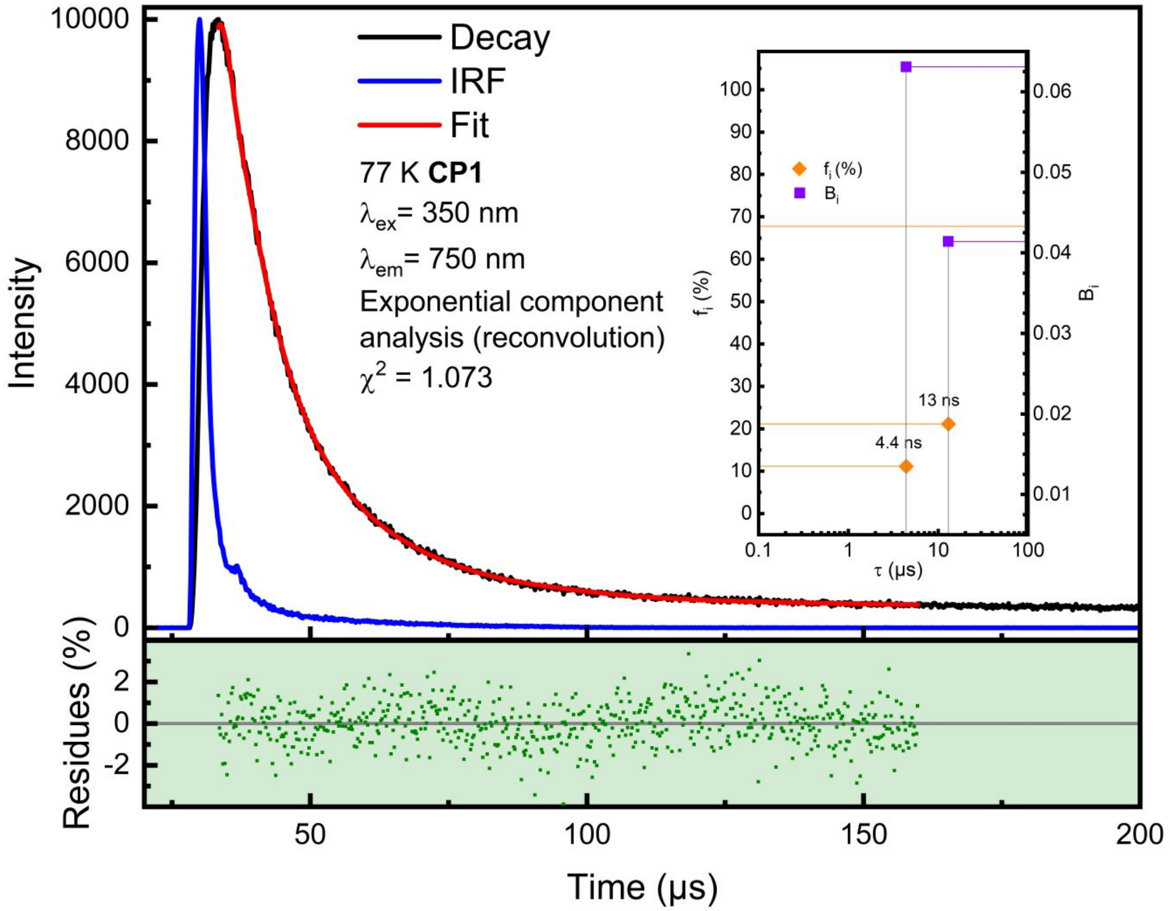

Figure S13. Photoluminescence decay curve of **CP1** at liquid nitrogen temperature. Depicted is the shorter part of the decay of the **B/C** band.

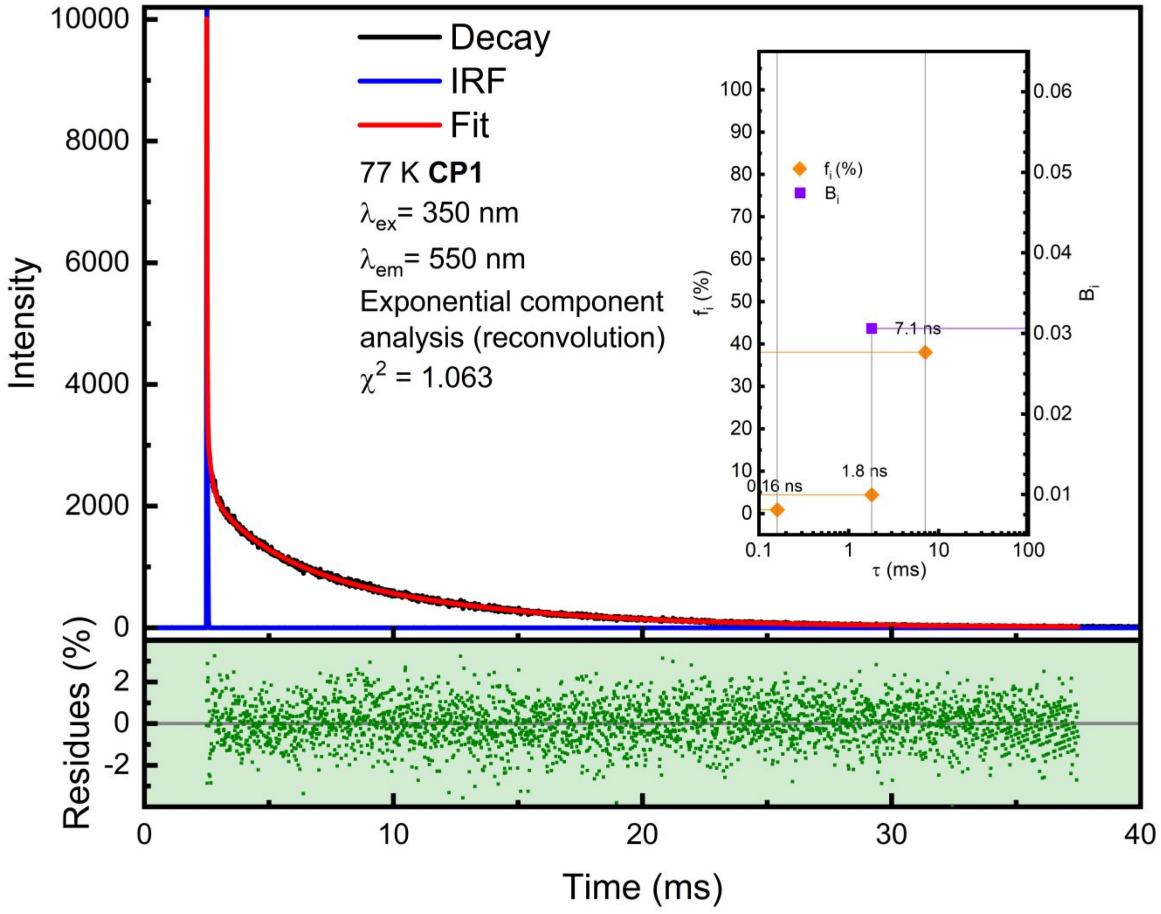

Figure S14. Photoluminescence decay curve of **CP1** at liquid nitrogen temeprature. Depicted is the longer part of the decay of the **A** band.

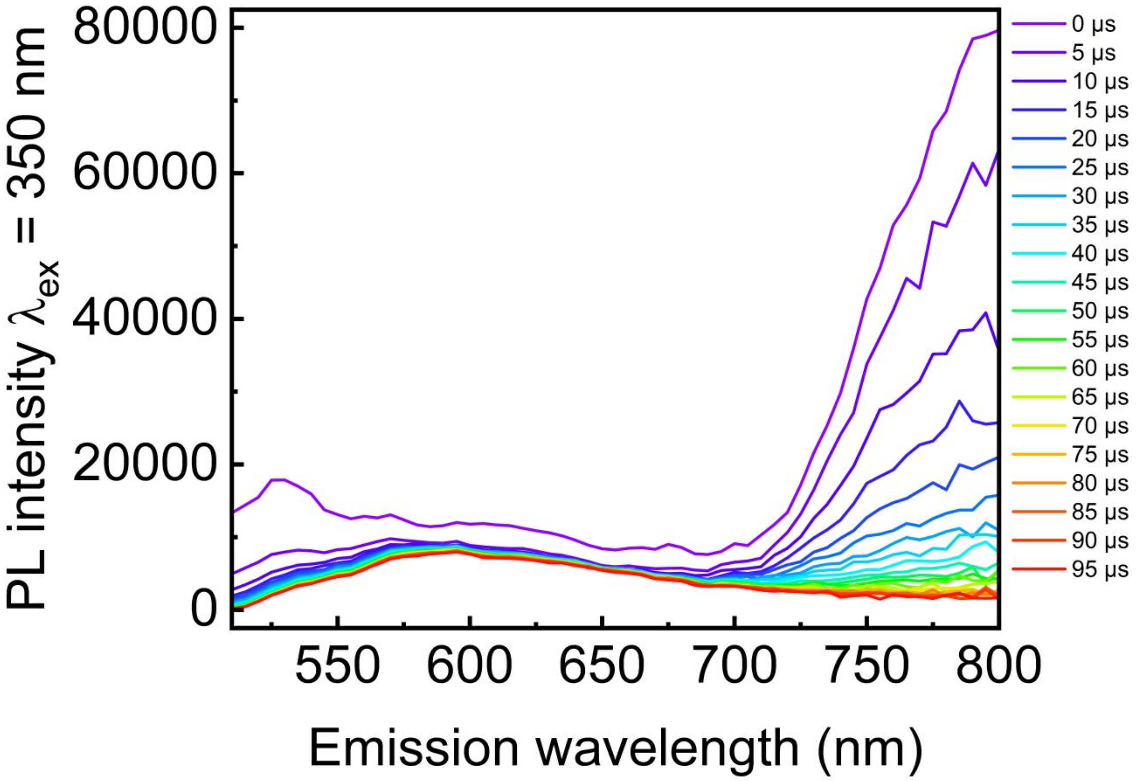

Figure S15. Absolute TREmS for **CP1** at liquid nitrogen temperature.

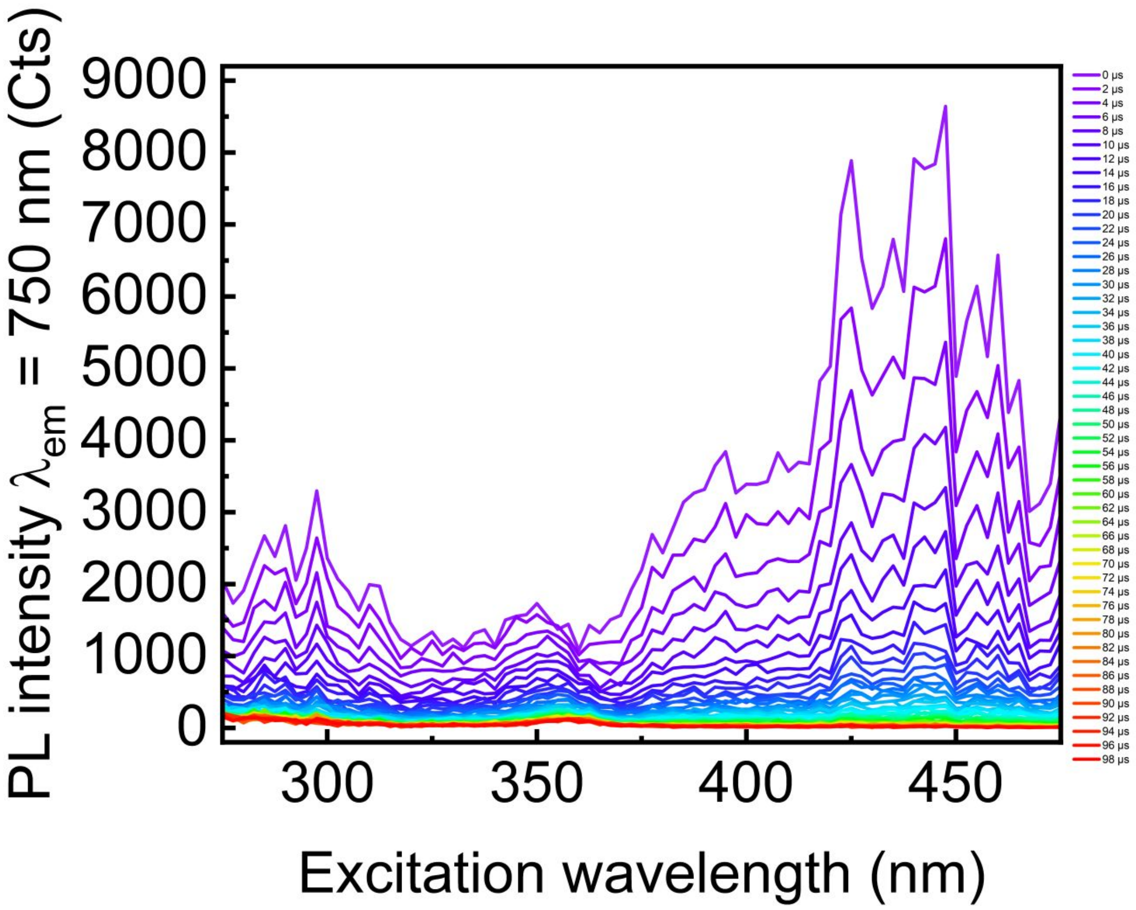

Figure S16. Absolute TRExS for **CP1** at liquid nitrogen temperature, measured at bands **B**/**C**.

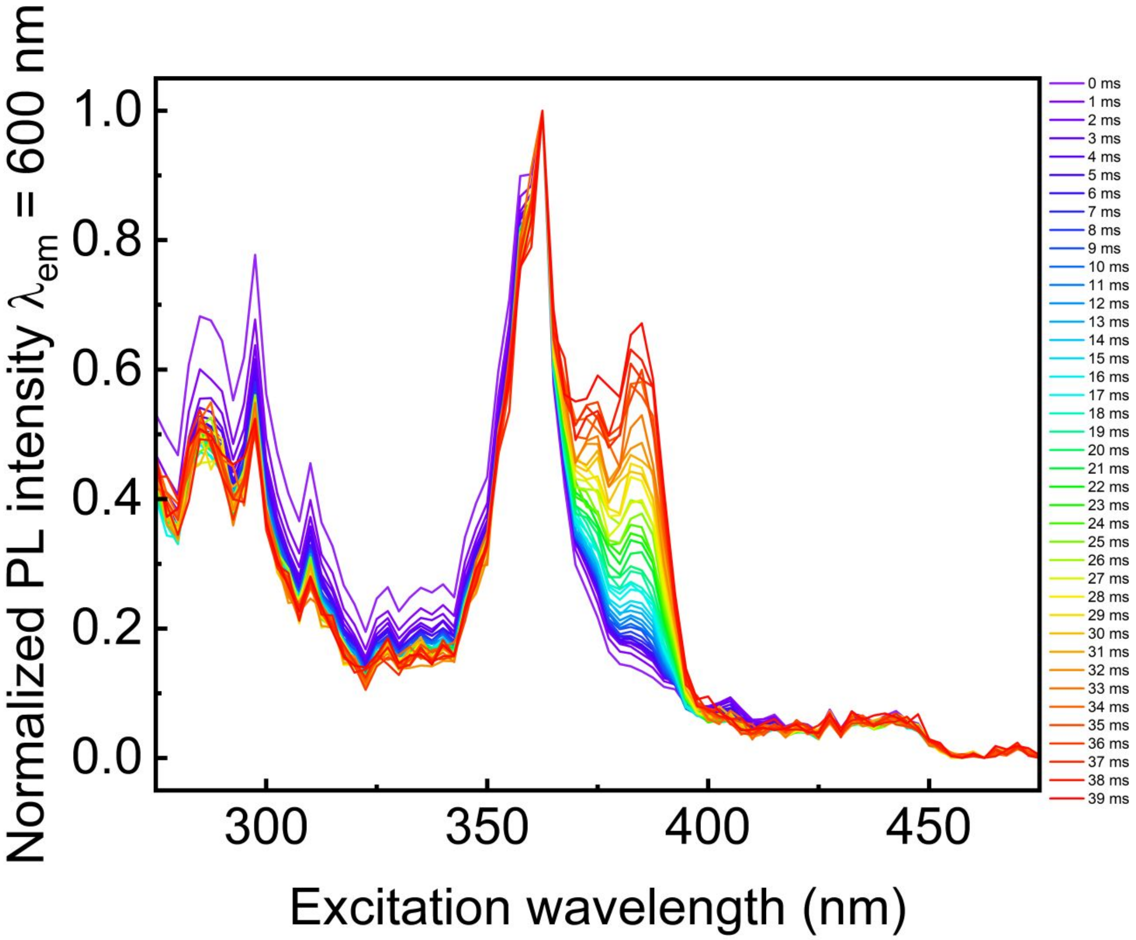

Figure S17. Normalized TRExS for **CP1** at liquid nitrogen temperature, measured at band **A**.

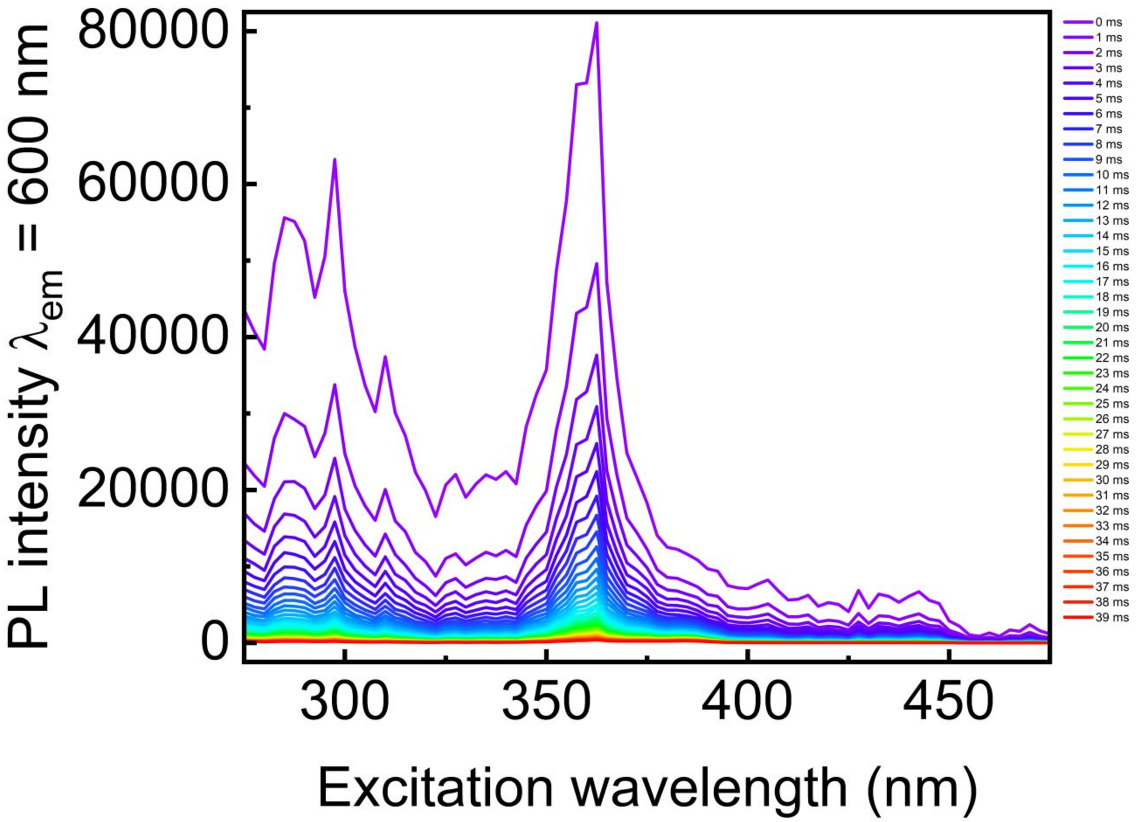

Figure S18. Absolute TRExS for **CP1** at liquid nitrogen temperature, measured at band **A**.

Table S10. Atomic coordinates for the optimized Cu_4I_4**L1**$_4$ conformer.

C	-0.12469752	4.34511124	-1.81101560
H	0.77358282	3.88027280	-2.21924571
H	-0.00346929	4.50907831	-0.73822431
C	0.47826737	5.75633328	-4.22294801
C	1.82239088	5.42149606	-4.21184972
H	2.34730695	5.30632449	-3.26754858
C	2.53422507	5.24453406	-5.42675719
H	3.58394410	4.96915058	-5.39194500
C	1.89813909	5.44386824	-6.63761068
H	2.44125105	5.32442717	-7.57185406
C	0.52654666	5.82253023	-6.68312410
C	-0.21255138	5.97785317	-5.45869754
C	-0.13243212	6.05241504	-7.92615432
H	0.43577393	5.93519916	-8.84599085
C	-1.46360969	6.42374316	-7.96697738
H	-1.95497578	6.60134272	-8.91946245
C	-2.19526823	6.57247377	-6.75753806
H	-3.24424879	6.85067861	-6.79392006
C	-1.58576952	6.35267228	-5.53546602
H	-2.15372101	6.45685717	-4.61672590
C	-1.57907597	2.76245488	-3.74072938
C	-0.50144015	2.12572425	-4.32854468
H	0.35143115	1.82243928	-3.72749028
C	-0.50171485	1.85972461	-5.72216394
H	0.36375229	1.37823198	-6.16681724
C	-1.59348730	2.20971886	-6.49418809
H	-1.59989375	2.00970246	-7.56290456
C	-2.72859059	2.83850547	-5.90913914
C	-2.73185523	3.13589966	-4.50205053
C	-3.86413543	3.18654236	-6.69603460
H	-3.85004556	2.95816586	-7.75902823
C	-4.96233368	3.80267633	-6.12577795
H	-5.82535477	4.05947946	-6.73343838
C	-4.96427496	4.10120581	-4.73565922
H	-5.82958009	4.58547487	-4.29209055
C	-3.87697172	3.77649613	-3.94378300
H	-3.88561965	4.00640720	-2.88346359
Cu	-0.54539855	1.23228971	-0.75182446
I	-2.13162293	-0.93301211	-1.32275843
S	-0.39939626	5.99738495	-2.62011217
S	-1.54560151	3.11200695	-1.93020179
Cu	1.29050176	0.38201878	1.26998151
Cu	-1.29050176	-0.38201878	1.26998151
Cu	0.54539855	-1.23228971	-0.75182446

I	-0.68044489	2.21961474	1.85345695
I	2.13162293	0.93301211	-1.32275843
C	3.36914988	2.75115945	2.34304097
H	2.77609017	3.11360075	3.18331603
H	2.87264598	3.00091299	1.40248401
C	3.11479470	0.57624659	4.23719145
C	1.95236907	1.03763049	4.82823729
H	1.19586435	1.54146111	4.23210900
C	1.72581104	0.83579169	6.21473831
H	0.81055544	1.21453010	6.65981940
C	2.65354799	0.14947553	6.97569239
H	2.48400162	-0.01349362	8.03747628
C	3.84138447	-0.36741674	6.38470809
C	4.09284420	-0.15428039	4.98438811
C	4.79051706	-1.09777579	7.15737646
H	4.59157502	-1.25394221	8.21507335
C	5.93817758	-1.60533202	6.57728982
H	6.65367290	-2.16449360	7.17346948
C	6.18517170	-1.39610748	5.19346905
H	7.09182679	-1.78914569	4.74371118
C	5.28676328	-0.68698382	4.41714114
H	5.48717976	-0.52706475	3.36310642
C	5.64461132	3.25303244	3.98936374
C	4.96427496	3.78419323	5.07224381
H	4.07773892	4.39085920	4.91022722
C	5.41862543	3.55225984	6.39619134
H	4.86035584	3.96548847	7.23063099
C	6.56889458	2.81720681	6.61423507
H	6.92769903	2.64208849	7.62536404
C	7.30619629	2.27997667	5.52183664
C	6.83940538	2.48535509	4.17704404
C	8.50189212	1.53505295	5.73665551
H	8.84605964	1.38652329	6.75736894
C	9.21338088	1.01047818	4.67386046
H	10.12700165	0.44932128	4.84835978
C	8.74736570	1.20429813	3.34452597
H	9.30695424	0.78509826	2.51309488
C	7.58934517	1.92227065	3.10262818
H	7.23321121	2.06362585	2.08724252
S	3.38220233	0.86677326	2.43191444
S	5.02670149	3.59095314	2.28838881
C	0.12469752	-4.34511124	-1.81101560
H	-0.77358282	-3.88027280	-2.21924571
H	0.00346929	-4.50907831	-0.73822431
C	-0.47826737	-5.75633328	-4.22294801
C	-1.82239088	-5.42149606	-4.21184972
H	-2.34730695	-5.30632449	-3.26754858

C	-2.53422507	-5.24453406	-5.42675719
H	-3.58394410	-4.96915058	-5.39194500
C	-1.89813909	-5.44386824	-6.63761068
H	-2.44125105	-5.32442717	-7.57185406
C	-0.52654666	-5.82253023	-6.68312410
C	0.21255138	-5.97785317	-5.45869754
C	0.13243212	-6.05241504	-7.92615432
H	-0.43577393	-5.93519916	-8.84599085
C	1.46360969	-6.42374316	-7.96697738
H	1.95497578	-6.60134272	-8.91946245
C	2.19526823	-6.57247377	-6.75753806
H	3.24424879	-6.85067861	-6.79392006
C	1.58576952	-6.35267228	-5.53546602
H	2.15372101	-6.45685717	-4.61672590
C	1.57907597	-2.76245488	-3.74072938
C	0.50144015	-2.12572425	-4.32854468
H	-0.35143115	-1.82243928	-3.72749028
C	0.50171485	-1.85972461	-5.72216394
H	-0.36375229	-1.37823198	-6.16681724
C	1.59348730	-2.20971886	-6.49418809
H	1.59989375	-2.00970246	-7.56290456
C	2.72859059	-2.83850547	-5.90913914
C	2.73185523	-3.13589966	-4.50205053
C	3.86413543	-3.18654236	-6.69603460
H	3.85004556	-2.95816586	-7.75902823
C	4.96233368	-3.80267633	-6.12577795
H	5.82535477	-4.05947946	-6.73343838
C	4.96427496	-4.10120581	-4.73565922
H	5.82958009	-4.58547487	-4.29209055
C	3.87697172	-3.77649613	-3.94378300
H	3.88561965	-4.00640720	-2.88346359
S	0.39939626	-5.99738495	-2.62011217
S	1.54560151	-3.11200695	-1.93020179
I	0.68044489	-2.21961474	1.85345695
C	-3.36914988	-2.75115945	2.34304097
H	-2.77609017	-3.11360075	3.18331603
H	-2.87264598	-3.00091299	1.40248401
C	-3.11479470	-0.57624659	4.23719145
C	-1.95236907	-1.03763049	4.82823729
H	-1.19586435	-1.54146111	4.23210900
C	-1.72581104	-0.83579169	6.21473831
H	-0.81055544	-1.21453010	6.65981940
C	-2.65354799	-0.14947553	6.97569239
H	-2.48400162	0.01349362	8.03747628
C	-3.84138447	0.36741674	6.38470809
C	-4.09284420	0.15428039	4.98438811
C	-4.79051706	1.09777579	7.15737646

H	-4.59157502	1.25394221	8.21507335
C	-5.93817758	1.60533202	6.57728982
H	-6.65367290	2.16449360	7.17346948
C	-6.18517170	1.39610748	5.19346905
H	-7.09182679	1.78914569	4.74371118
C	-5.28676328	0.68698382	4.41714114
H	-5.48717976	0.52706475	3.36310642
C	-5.64461132	-3.25303244	3.98936374
C	-4.96427496	-3.78419323	5.07224381
H	-4.07773892	-4.39085920	4.91022722
C	-5.41862543	-3.55225984	6.39619134
H	-4.86035584	-3.96548847	7.23063099
C	-6.56889458	-2.81720681	6.61423507
H	-6.92769903	-2.64208849	7.62536404
C	-7.30619629	-2.27997667	5.52183664
C	-6.83940538	-2.48535509	4.17704404
C	-8.50189212	-1.53505295	5.73665551
H	-8.84605964	-1.38652329	6.75736894
C	-9.21338088	-1.01047818	4.67386046
H	-10.12700165	-0.44932128	4.84835978
C	-8.74736570	-1.20429813	3.34452597
H	-9.30695424	-0.78509826	2.51309488
C	-7.58934517	-1.92227065	3.10262818
H	-7.23321121	-2.06362585	2.08724252
S	-3.38220233	-0.86677326	2.43191444
S	-5.02670149	-3.59095314	2.28838881

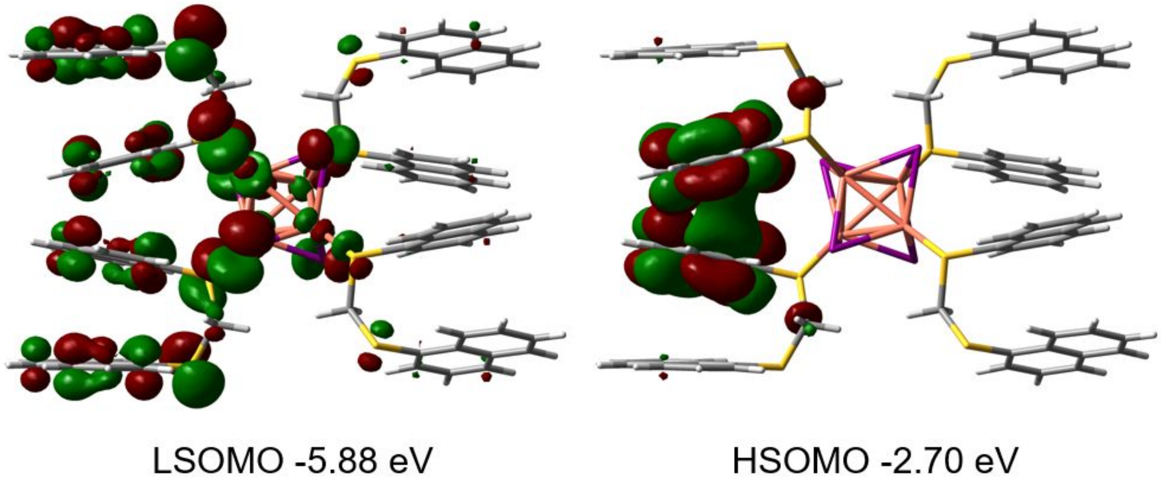

Figure S19. Frontier molecular orbitals in the triplet excited state for Cu_4I_4**L1**$_4$.

Table S11. Atomic coordinates of the Cu_4I_4**L1**$_4$ fragment in its optimized triplet excited state.

C	4.15675709	-1.21819293	-2.15937342
H	3.44461493	-1.94495401	-2.55208460
H	4.27882748	-1.36175129	-1.08385331
C	5.31969659	-2.26034534	-4.55390669
C	4.60474976	-3.44629671	-4.51813620
H	4.34430217	-3.89634208	-3.56410708
C	4.22228769	-4.09734475	-5.71976322
H	3.65115079	-5.01923086	-5.66604024
C	4.59544677	-3.57039489	-6.94189463
H	4.31862154	-4.07181356	-7.86599671
C	5.36007334	-2.37162666	-7.01245931
C	5.73005677	-1.68811009	-5.80186845
C	5.76913344	-1.83264378	-8.26720858
H	5.48706467	-2.35838365	-9.17643225
C	6.51520965	-0.67046201	-8.33225997
H	6.82618421	-0.27091622	-9.29341021
C	6.87667782	0.00758420	-7.13651423
H	7.45100225	0.92742762	-7.19213049
C	6.49160888	-0.48737664	-5.90363956
H	6.76151118	0.04171993	-4.99552478
C	3.06260288	0.59678966	-4.12106488
C	2.13161933	-0.25874949	-4.68116813
H	1.59441967	-0.96899230	-4.05841370
C	1.86757795	-0.21409484	-6.07443665
H	1.14688063	-0.90720604	-6.49703030
C	2.51954602	0.70594599	-6.87388578
H	2.32298921	0.74443054	-7.94256902
C	3.45956365	1.61884890	-6.31785919
C	3.75418242	1.56922591	-4.91106571
C	4.12163239	2.58143752	-7.13324063
H	3.89269728	2.60871791	-8.19584701
C	5.03725822	3.46339862	-6.59046130
H	5.53276044	4.19723962	-7.21967073
C	5.33216898	3.41195060	-5.20051796
H	6.05228788	4.10729028	-4.77875714
C	4.70728459	2.48814425	-4.38148531
H	4.93673275	2.45474397	-3.32154770
Cu	1.33027301	0.14229792	-1.08741135
I	-0.23271386	2.33969976	-1.57836494
S	5.81415468	-1.46373318	-2.96685537
S	3.40264836	0.50327573	-2.31079535
Cu	0.00031778	-1.32620360	0.99194109
Cu	-0.00031778	1.32620360	0.99194109
Cu	-1.33027301	-0.14229792	-1.08741135

I	2.35848998	0.01837126	1.48527739
I	0.23271386	-2.33969976	-1.57836494
C	1.60082548	-3.95674482	2.18864795
H	2.26493007	-3.18297864	2.57481056
H	1.75774096	-4.08371848	1.11515678
C	-0.29708015	-2.87385130	4.09175202
C	0.57570103	-1.88753906	4.60286555
H	1.29044024	-1.40133196	3.94630003
C	0.53059783	-1.54119908	5.95428336
H	1.22553124	-0.80259927	6.33882868
C	-0.40422943	-2.15519111	6.81273691
H	-0.43420873	-1.89248535	7.86721482
C	-1.32847164	-3.12087457	6.31614127
C	-1.28785343	-3.50088981	4.92305943
C	-2.29007887	-3.72343013	7.16504746
H	-2.30816026	-3.44172467	8.21517177
C	-3.20264391	-4.67139843	6.67631031
H	-3.93508960	-5.11624486	7.34359589
C	-3.16341479	-5.03882654	5.32440886
H	-3.86759694	-5.76921848	4.93661539
C	-2.21498371	-4.46398600	4.45903822
H	-2.19030744	-4.76281652	3.41585355
C	2.48148579	-5.02334145	4.68719596
C	3.57877555	-4.18357687	4.80134172
H	4.10870257	-3.85718631	3.91068245
C	4.03629847	-3.76053488	6.07504293
H	4.89082632	-3.09373825	6.13865908
C	3.41026240	-4.21467155	7.22130313
H	3.76555139	-3.90731331	8.20179544
C	2.29844201	-5.10048809	7.13997420
C	1.80644858	-5.51284068	5.85269683
C	1.65863068	-5.58669890	8.31674698
H	2.04045643	-5.27172882	9.28505069
C	0.57570103	-6.44273200	8.23492448
H	0.09599218	-6.80888276	9.13811966
C	0.08253964	-6.84152178	6.96361927
H	-0.77965565	-7.49887962	6.90361014
C	0.68188032	-6.38678606	5.80237948
H	0.29232160	-6.68555789	4.83468294
S	-0.18439383	-3.34000526	2.33083901
S	1.95496627	-5.58095992	3.01404337
C	-4.15675709	1.21819293	-2.15937342
H	-3.44461493	1.94495401	-2.55208460
H	-4.27882748	1.36175129	-1.08385331
C	-5.31969659	2.26034534	-4.55390669
C	-4.60474976	3.44629671	-4.51813620
H	-4.34430217	3.89634208	-3.56410708

C	-4.22228769	4.09734475	-5.71976322
H	-3.65115079	5.01923086	-5.66604024
C	-4.59544677	3.57039489	-6.94189463
H	-4.31862154	4.07181356	-7.86599671
C	-5.36007334	2.37162666	-7.01245931
C	-5.73005677	1.68811009	-5.80186845
C	-5.76913344	1.83264378	-8.26720858
H	-5.48706467	2.35838365	-9.17643225
C	-6.51520965	0.67046201	-8.33225997
H	-6.82618421	0.27091622	-9.29341021
C	-6.87667782	-0.00758420	-7.13651423
H	-7.45100225	-0.92742762	-7.19213049
C	-6.49160888	0.48737664	-5.90363956
H	-6.76151118	-0.04171993	-4.99552478
C	-3.06260288	-0.59678966	-4.12106488
C	-2.13161933	0.25874949	-4.68116813
H	-1.59441967	0.96899230	-4.05841370
C	-1.86757795	0.21409484	-6.07443665
H	-1.14688063	0.90720604	-6.49703030
C	-2.51954602	-0.70594599	-6.87388578
H	-2.32298921	-0.74443054	-7.94256902
C	-3.45956365	-1.61884890	-6.31785919
C	-3.75418242	-1.56922591	-4.91106571
C	-4.12163239	-2.58143752	-7.13324063
H	-3.89269728	-2.60871791	-8.19584701
C	-5.03725822	-3.46339862	-6.59046130
H	-5.53276044	-4.19723962	-7.21967073
C	-5.33216898	-3.41195060	-5.20051796
H	-6.05228788	-4.10729028	-4.77875714
C	-4.70728459	-2.48814425	-4.38148531
H	-4.93673275	-2.45474397	-3.32154770
S	-5.81415468	1.46373318	-2.96685537
S	-3.40264836	-0.50327573	-2.31079535
I	-2.35848998	-0.01837126	1.48527739
C	-1.60082548	3.95674482	2.18864795
H	-2.26493007	3.18297864	2.57481056
H	-1.75774096	4.08371848	1.11515678
C	0.29708015	2.87385130	4.09175202
C	-0.57570103	1.88753906	4.60286555
H	-1.29044024	1.40133196	3.94630003
C	-0.53059783	1.54119908	5.95428336
H	-1.22553124	0.80259927	6.33882868
C	0.40422943	2.15519111	6.81273691
H	0.43420873	1.89248535	7.86721482
C	1.32847164	3.12087457	6.31614127
C	1.28785343	3.50088981	4.92305943
C	2.29007887	3.72343013	7.16504746

H	2.30816026	3.44172467	8.21517177
C	3.20264391	4.67139843	6.67631031
H	3.93508960	5.11624486	7.34359589
C	3.16341479	5.03882654	5.32440886
H	3.86759694	5.76921848	4.93661539
C	2.21498371	4.46398600	4.45903822
H	2.19030744	4.76281652	3.41585355
C	-2.48148579	5.02334145	4.68719596
C	-3.57877555	4.18357687	4.80134172
H	-4.10870257	3.85718631	3.91068245
C	-4.03629847	3.76053488	6.07504293
H	-4.89082632	3.09373825	6.13865908
C	-3.41026240	4.21467155	7.22130313
H	-3.76555139	3.90731331	8.20179544
C	-2.29844201	5.10048809	7.13997420
C	-1.80644858	5.51284068	5.85269683
C	-1.65863068	5.58669890	8.31674698
H	-2.04045643	5.27172882	9.28505069
C	-0.57570103	6.44273200	8.23492448
H	-0.09599218	6.80888276	9.13811966
C	-0.08253964	6.84152178	6.96361927
H	0.77965565	7.49887962	6.90361014
C	-0.68188032	6.38678606	5.80237948
H	-0.29232160	6.68555789	4.83468294
S	0.18439383	3.34000526	2.33083901
S	-1.95496627	5.58095992	3.01404337

Table S12. List of the first singlet-triplet transitions in the Cu_4I_4**L1**$_4$ fragment (S_0 wavefunction, ^{3}M/XLCT geometry). Since the calculations did not include spin-orbit coupling, all oscillator strengths are nil.

N°	λ (nm)	Osc. str.	Major contributions
1	574	0.0000	H-10→L+1 (10%), H-1→L+1 (12%), HOMO→LUMO (26%)
2	570	0.0000	H-10→LUMO (12%), H-1→LUMO (16%), HOMO→L+1 (20%)
3	500	0.0000	H-7→L+5 (16%), HOMO→L+5 (11%)
4	500	0.0000	H-7→L+7 (13%), H-1→L+5 (11%), HOMO→L+7 (10%)
5	496	0.0000	H-6→L+4 (12%), H-3→L+3 (12%)

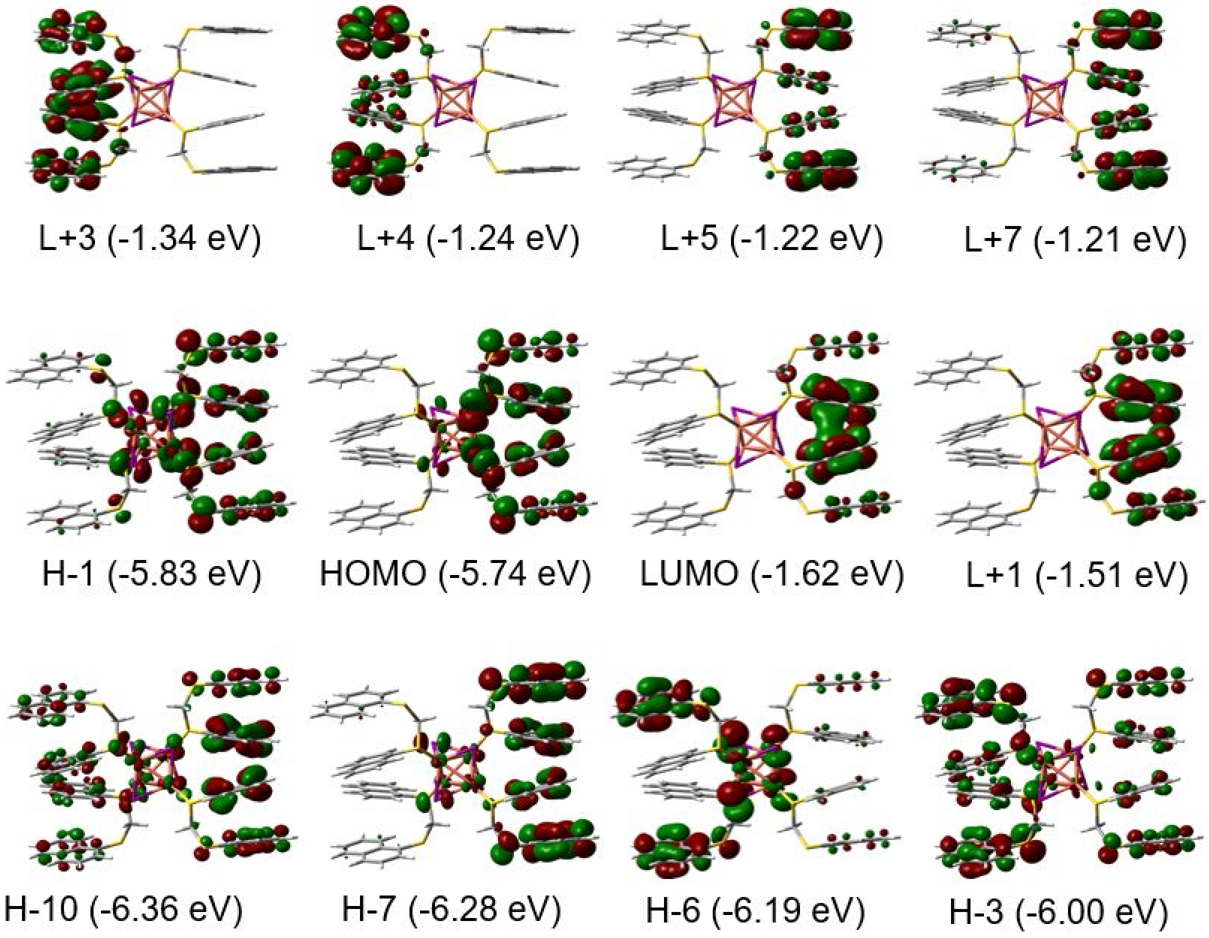

Figure S20. Representation of the frontier molecular orbitals of the Cu_4I_4**L1**$_4$ fragment in its ^{3}M/XLCT geometry.

Table S13. List of the first 30 spin-allowed electronic transitions in the Cu_4I_4**L1**$_4$ fragment. Due to disk memory limitations, the list is limited to these transitions and could not be extended to lower wavelengths.

N°	λ (nm)	Osc. str.	Major contributions
1	331	0.0188	H-2→L+3 (12%), H-1→L+1 (20%), HOMO→LUMO (11%), HOMO→L+1 (47%)
2	330	0.0397	H-1→LUMO (47%), HOMO→LUMO (22%)
3	328	0.0038	H-2→L+1 (29%), H-1→L+3 (14%), HOMO→L+3 (37%)
4	327	0.0121	H-2→LUMO (20%), H-1→L+2 (36%), HOMO→L+2 (21%)
5	314	0.0487	H-2→L+2 (13%), H-1→L+4 (34%), HOMO→L+4 (24%)
6	312	0.1210	H-2→LUMO (20%), H-2→L+4 (18%), H-1→L+5 (18%), HOMO→L+5 (10%)
7	311	0.0439	HOMO→LUMO (17%), HOMO→L+6 (31%)
8	311	0.0118	H-1→LUMO (20%), HOMO→LUMO (35%), HOMO→L+6 (13%)
9	310	0.0244	H-2→L+1 (23%), H-2→L+6 (16%), HOMO→L+3 (19%), HOMO→L+7 (14%)
10	310	0.0384	H-2→LUMO (36%), H-1→L+5 (15%), HOMO→L+2 (10%), HOMO→L+5 (16%)
11	309	0.0014	H-1→L+1 (51%), HOMO→L+1 (27%)
12	308	0.0273	H-2→L+1 (32%), HOMO→L+3 (11%), HOMO→L+7 (29%)
13	307	0.0026	H-4→L+1 (12%), H-2→L+3 (26%), H-1→L+1 (14%)
14	306	0.0078	H-2→L+2 (26%), H-2→L+5 (11%), HOMO→L+8 (19%)
15	306	0.0023	H-2→L+2 (12%), HOMO→L+8 (40%)
16	306	0.0044	H-1→L+2 (37%), HOMO→L+2 (41%)
17	305	0.0080	H-6→L+8 (13%), H-1→L+8 (67%)
18	304	0.0364	H-5→L+1 (16%), H-4→L+3 (14%), H-3→L+1 (34%)
19	303	0.0010	H-1→L+3 (60%), HOMO→L+3 (13%)
20	303	0.0077	H-5→L+3 (11%), H-4→L+1 (18%), H-3→L+3 (11%), H-2→L+3 (27%)
21	303	0.0014	H-5→L+8 (13%), H-2→L+8 (52%), H-1→L+3 (13%)
22	302	0.0037	H-6→L+2 (12%), H-5→LUMO (26%), H-3→LUMO (21%)
23	301	0.0648	H-6→LUMO (26%), H-5→L+2 (17%)
24	295	0.0001	H-1→L+4 (23%), HOMO→L+4 (63%)
25	294	0.0000	H-2→L+4 (16%), H-1→L+5 (10%), HOMO→L+5 (60%)
26	293	0.0015	H-7→LUMO (19%), H-4→LUMO (45%)
27	292	0.0018	H-2→L+4 (48%), H-1→L+5 (37%)
28	292	0.0016	H-7→L+1 (12%), H-4→L+1 (36%)
29	292	0.0027	H-2→L+5 (49%), H-1→L+4 (24%)
30	291	0.0019	H-2→L+7 (23%), H-1→L+6 (13%), HOMO→L+6 (45%)

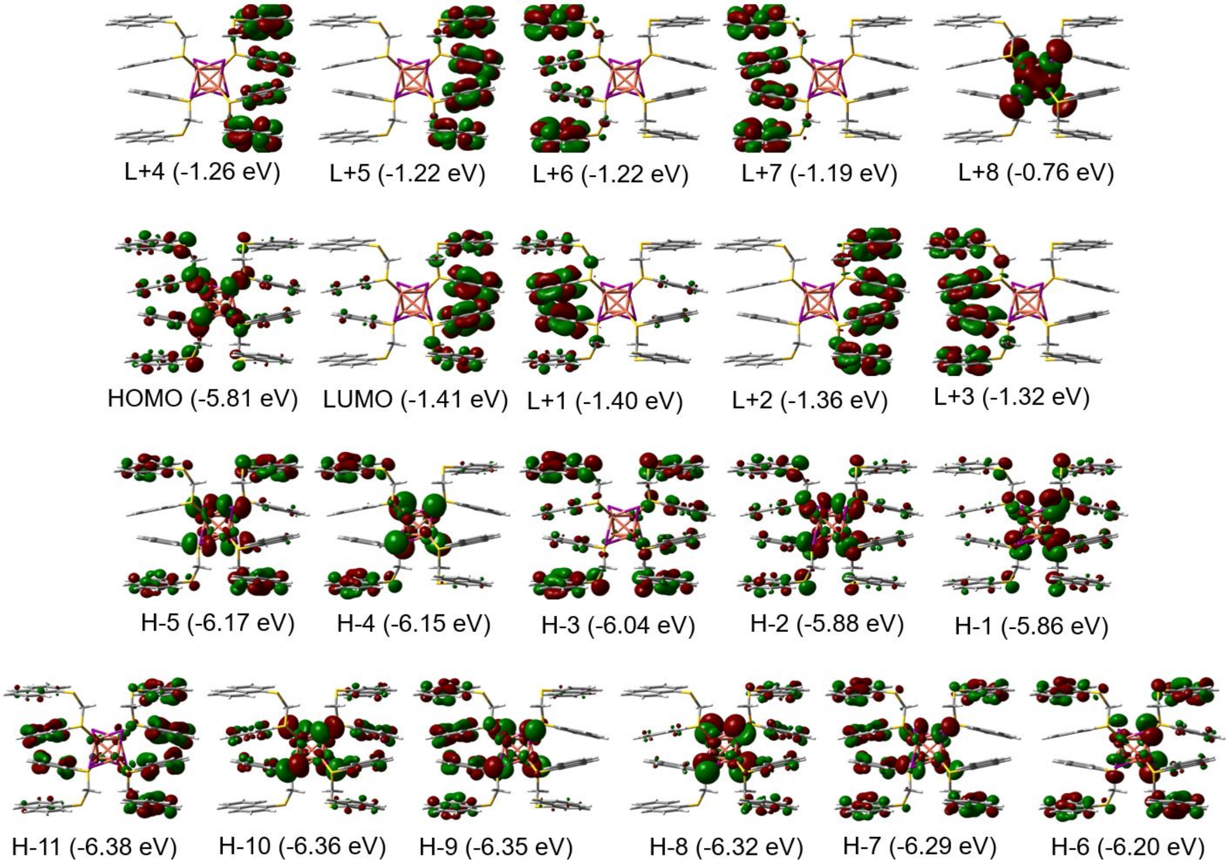

Figure S21. Representation of relevant molecular orbitals in the Cu_4I_4**L1**$_4$ fragment (ground state).

Table S14. List of the first 10 singlet-triplet transitions in Cu_4I_4**L1**$_4$ (S_0). Since the calculations did not include spin-orbit coupling, all oscillator strengths are nil. Relevant molecular orbitals are depicted in **Figure S21**.

N°	λ (nm)	Osc. str.	Major contributions
1	496	0.0000	H-3→L+3 (10%)
2	496	0.0000	H-3→L+6 (11%)
3	495	0.0000	H-3→L+2 (11%)
4	495	0.0000	H-11→L+4 (10%)
5	491	0.0000	H-11→L+2 (11%)
6	490	0.0000	H-11→LUMO (13%)
7	490	0.0000	*Only minor contributions are observed*
8	490	0.0000	H-9→L+1 (10%)
9	336	0.0000	H-1→LUMO (14%), HOMO→LUMO (17%)
10	336	0.0000	H-1→LUMO (10%), HOMO→L+1 (26%)

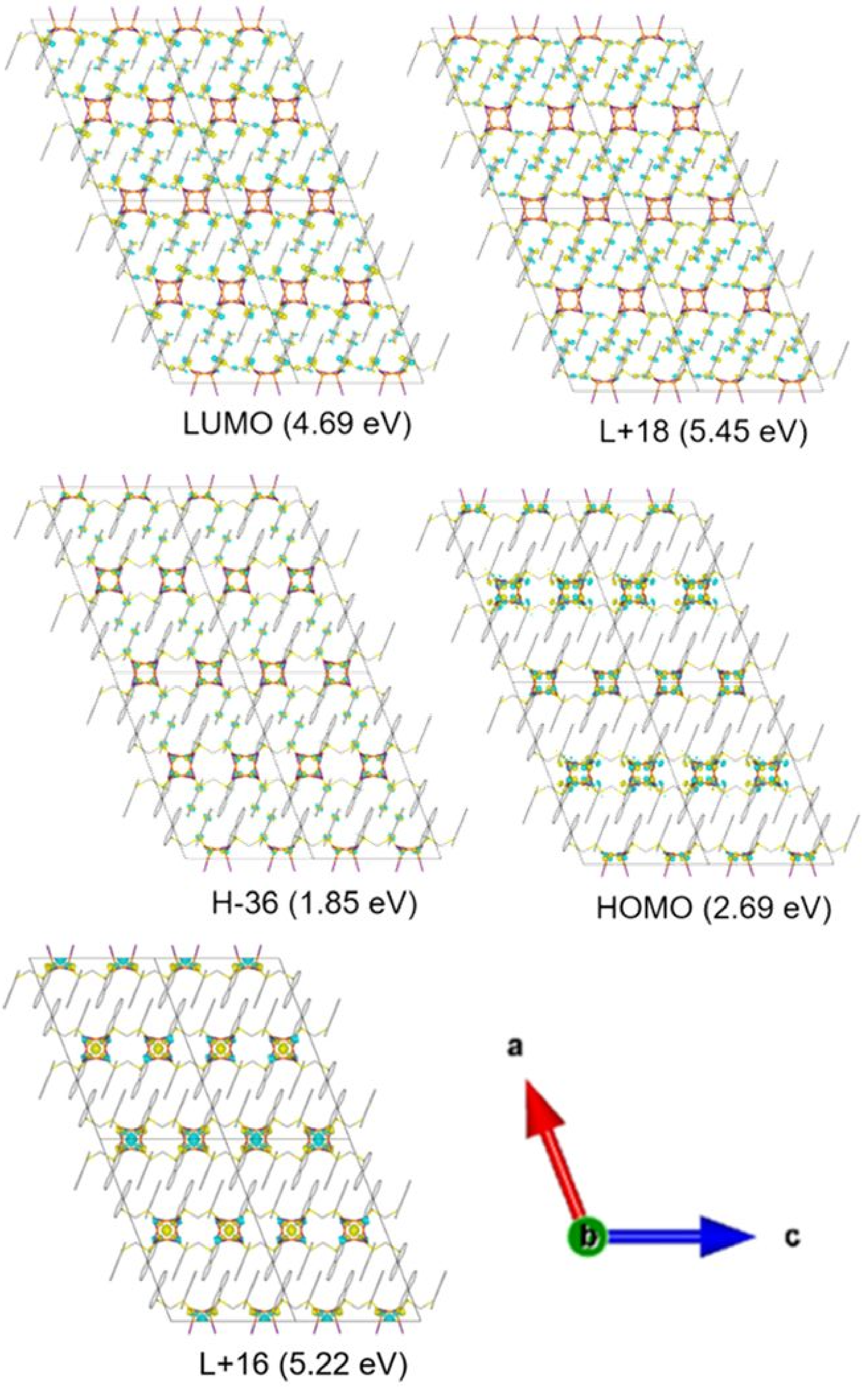

Figure S22. Representation of relevant solid-state molecular orbitals of **CP1**.

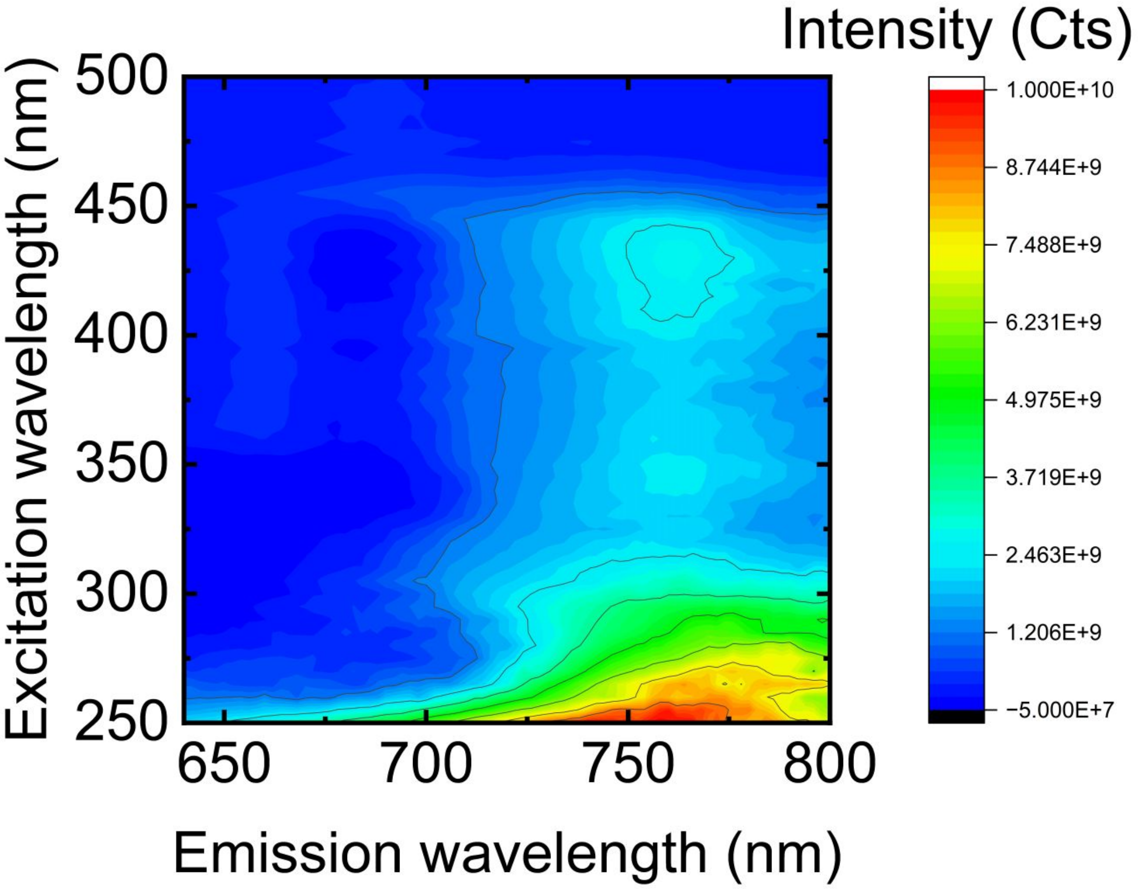

Figure S23. Excitation-emission map of **L1** in bromopropane solution at 77 K.
